# Prevalence and risk factors associated with birth asphyxia among neonates delivered in Ethiopia: A systematic review and meta-analysis

**DOI:** 10.1371/journal.pone.0255488

**Published:** 2021-08-05

**Authors:** Ritbano Ahmed, Hassen Mosa, Mohammed Sultan, Shamill Eanga Helill, Biruk Assefa, Muhammed Abdu, Usman Ahmed, Selamu Abose, Amanuel Nuramo, Abebe Alemu, Minychil Demelash, Romedan Delil

**Affiliations:** 1 Department of Midwifery, College of Medicine and Health Science, Wachemo University, Hossana, Ethiopia; 2 Department of Statistics, Collage of Natural and Computational Science, Wachemo University, Hosanna, Ethiopia; 3 Department of Anesthesia, College of Medicine and Health Sciences, Wachemo University, Hossana, Ethiopia; 4 Department of Midwifery, College of Health Sciences, Samara University, Samara, Ethiopia; 5 Department of Nursing, College of Health Sciences, Samara University, Samara, Ethiopia; 6 Department of Nursing, Hossana College of Health Science, Hossana, Ethiopia; University of Oklahoma, UNITED STATES

## Abstract

**Background:**

A number of primary studies in Ethiopia address the prevalence of birth asphyxia and the factors associated with it. However, variations were seen among those studies. The main aim of this systematic review and meta-analysis was carried out to estimate the pooled prevalence and explore the factors that contribute to birth asphyxia in Ethiopia.

**Methods:**

Different search engines were used to search online databases. The databases include PubMed, HINARI, Cochrane Library and Google Scholar. Relevant grey literature was obtained through online searches. The funnel plot and Egger’s regression test were used to see publication bias, and the I-squared was applied to check the heterogeneity of the studies. Cross-sectional, case-control and cohort studies that were conducted in Ethiopia were also be included. The Joanna Briggs Institute checklist was used to assess the quality of the studies and was included in this systematic review. Data entry and statistical analysis were carried out using RevMan 5.4 software and Stata 14.

**Result:**

After reviewing 1,125 studies, 26 studies fulfilling the inclusion criteria were included in the meta-analysis. The pooled prevalence of birth asphyxia in Ethiopia was 19.3%. In the Ethiopian context, the following risk factors were identified: Antepartum hemorrhage(OR: 4.7; 95% CI: 3.5, 6.1**)**, premature rupture of membrane(OR: 4.0; 95% CI: 12.4, 6.6), primiparas(OR: 2.8; 95% CI: 1.9, 4.1), prolonged labor(OR: 4.2; 95% CI: 2.8, 6.6), maternal anaemia(OR: 5.1; 95% CI: 2.59, 9.94), low birth weight(OR = 5.6; 95%CI: 4.7,6.7), meconium stained amniotic fluid(OR: 5.6; 95% CI: 4.1, 7.5), abnormal presentation(OR = 5.7; 95% CI: 3.8, 8.3), preterm birth(OR = 4.1; 95% CI: 2.9, 5.8), residing in a rural area (OR: 2.7; 95% CI: 2.0, 3.5), caesarean delivery(OR = 4.4; 95% CI:3.1, 6.2), operative vaginal delivery(OR: 4.9; 95% CI: 3.5, 6.7), preeclampsia(OR = 3.9; 95% CI: 2.1, 7.4), tight nuchal cord OR: 3.43; 95% CI: 2.1, 5.6), chronic hypertension(OR = 2.5; 95% CI: 1.7, 3.8), and unable to write and read (OR = 4.2;95%CI: 1.7, 10.6).

**Conclusion:**

According to the findings of this study, birth asphyxia is an unresolved public health problem in the Ethiopia. Therefore, the concerned body needs to pay attention to the above risk factors in order to decrease the country’s birth asphyxia.

**Review registration:**

PROSPERO International prospective register of systematic reviews (CRD42020165283).

## Introduction

Birth asphyxia, which is defined as the failure to establish breathing at birth [1], accounts for an estimated 600,000 neonatal deaths worldwide (24%) each year and is one of the primary causes of early neonatal mortality [2,3]. According to the International Classification of Diseases, Tenth Revision (ICD-10) of the World Health Organization [WHO], severe birth asphyxia is when the Apgar score at 1 min is 0–3. Mild and moderate birth asphyxia is when Apgar score at 1 min is 4–7 [4]. Birth asphyxia comprises a larger proportion of cases in developing countries than their developed counterparts due to the reduced availability of skilled care during delivery [3,5]. The condition may be caused by antepartum, intrapartum/postpartum events, or a combination of these [4,6].

Numerous conditions can predispose a baby to birth asphyxia, but the underlying aetiology is decreased cerebral blood flow [7]. These include maternal events (hemorrhage, amniotic fluid embolism or high blood pressure), placental events (acute abruption), uterine events (rupture), cord events (tight nuchal cord or cord prolapse/avulsion), intrapartum infection, and very long or difficult delivery [7,8].

The impact of birth asphyxia ranges from being mild to severe including HIE, multi-organ dysfunction and death, with a dose-response gradient between severity of asphyxia and the ensuing complications. HIE is one of the most important causes for severe neurodevelopmental and motor delay, which become increasingly apparent as the child develops [9,10].

The Government of Ethiopia has developed different strategies for addressing this. These strategies include an increase in coverage of antenatal care, skilled attendance and post-natal care [11]. The guidelines for treating birth asphyxia are also well established in Ethiopia and are even made available at the health centres, for assessment and classification [12].

Nevertheless, the number of deaths that are due to birth asphyxia remains very high in Ethiopia [13,14]. Studies conducted in Ethiopia, in Tigray [15] and Jimma [16], revealed that birth asphyxia was responsible for 35% and 47.5% of neonatal deaths, respectively. Furthermore, several primary studies conducted in the different regions of the country have confirmed that prevalence varies from one region to the next and that various factors are associated with birth asphyxia [17–22].

A nationwide study (in the Ethiopian context) has not yet been conducted. In order to improve intervention, it is vital to acquire up-to-dated information about the prevalence of the condition and its associated factors. The main aims of this systematic review and meta-analysis are therefore to estimate pooled prevalence of birth asphyxia and the associated factors in the Ethiopian context, thereby contributing to the data available for decision-makers.

## Methods

This systematic review and meta-analysis was reported using the Preferred Reporting Items for Systematic Reviews and Meta-Analyses (PRISMA) [23] guidelines to determine the pooled prevalence and risk factors associated with birth asphyxia in Ethiopia. This systematic review was developed based on the PRISMA checklist as presented in **S1 File**. The review protocol was developed based on the Preferred Reporting Items for Systematic Reviews and Meta-Analyses (PRISMA) 2015 [24]. It was then submitted and published in PROSPERO International prospective register of systematic reviews with the ID = CRD42020165283; available at http://www.crd.york.ac.uk/PROSPERO).

### Inclusion criteria

#### Study area

Ethiopia.

#### Study participants

Newborns and their mothers.

#### Types of studies

Cross–sectional and case-control studies were included.

#### Outcome of interests

These studies reported the prevalence and/or at least one risk factors for birth asphyxia were considered.

#### Publication condition

All published articles and gray literatures.

#### Language

English language.

#### Publication date

Only studies were conducted in Ethiopia from 25 November 2009 up to 25 November 2020, due to the lack of studies from Ethiopia on the topic before 2009.

### Exclusion criteria

Articles without abstract and/or full-text, duplicated studies, anonymous reports, editorials, and qualitative studies were excluded from the analysis. In addition, studies that did not include events in both the risk and non-risk groups were excluded after at least two email contacts of the primary author. Exclusion of these studies were because of inability to extract the data of studies in the absence of hard data.

### Outcome measurement

This systematic review has two outcomes. Birth asphyxia, as the primary outcome variable of this study, is defined as the failure to establish breathing at birth [1]. The pooled prevalence of birth asphyxia (5^th^ minute Apgar score less than 7) was calculated from the prevalence in each primary study. The second outcomes were the risk factors of birth asphyxia. The pooled odds ratio (AOR) was calculated based on the primary studies that studied the relationship between each factor with birth asphyxia.

**Prolonged labor**:Prolonged if it exceeds 12 hrs after onset of true labour**. Preeclampsia** is defined as the sudden onset of hypertension and proteinuria in a previously normotensive woman after 20 weeks of gestation**. Chronic hypertension:** Hypertension that antedates pregnancy; is present before 20 weeks of gestation; or persists after 12 weeks postpartum. **Complications during Labor:** Malpresentation, malposition, prolonged labour or obstructed labour, or/and others. **Tight nuchal cord ‘**was defined as the inability to manually reduce the loop over the fetal head. **Low birth weight**: is defined as the **birth weight** of an infant of 2,499 g (5 lb 8.1 oz) or less, regardless of gestational age. Prolonged second stage labor: Prolonged if it exceeds 3.5 h with provision of regional anaesthesia, or 2 h in the absence of regional anaesthesia for nulliparas. It is multiparous if it exceeds 2.5 h with regional anaesthesia or 1 h without it. We have assessed this in numbers. **Ante-partum hemorrhage**: Vaginal bleeding from the 28th week of gestation till the fetus (last fetus in case of multiple pregnancies) is delivered. **Abnormal presentations**: Are all presentations other than vertex. **Preterm:** Babies born alive before 37 weeks of pregnancy are completed.

### Search strategy

The international databases, including PubMed, Medline, Hinari, Cochrane library and Google Scholar were systematically searched. Relevant grey literature was also obtained through online searches. The search was conducted using the following keywords: prevalence, incidence, magnitude, asphyxia, birth asphyxia, perinatal asphyxia, Apgar score, hypoxic-ischemic encephalopathy, asphyxia neonatorum, risk factors, factors, predictors, correlates, determinants, contributing factors, neonates, infant, newborn, and Ethiopia. The search terms were used separately and in combination with Boolean operators such as ‘OR’ or ‘AND’. All papers published and unpublished from 25 November 2009 to 25 November 2020 were included in this review. The specific electronic search strategy is provided in **S2 File**.

### Data extraction

After collecting findings from all databases, the articles were exported to Endnote X7. Two authors (RA and HM) independently extracted all necessary data using a standardized data extraction form. Any disagreements at the time of data extraction were handled by the third reviewer (MS). The data extraction format included primary author and publication year, study area, study design, sample size, response rate, prevalence/incidence and risk factors and the quality score of each study.

#### Quality assessment/Critical appraisal

The quality of individual study was appraised using the Joanna Briggs Institute (JBI) quality appraisal Checklist [25]. Two authors independently assessed the quality of each primary study. Disagreements between the two authors were resolved by taking the mean score of the two authors. A quality was considered “low risk” if a study awarded ≥50% of the quality assessment indicators. Cross-sectional studies were appraised using eight items: (1) inclusion criteria, (2) description of study subject and setting, (3) valid and reliable measurement of exposure, (4) objective and standard criteria used, (5) identification of confounder, (5) strategies to handle confounder, (7) outcome measurement, and (8) appropriate statistical analysis. Case–control studies were appraised using 10 items: (1) comparable groups, (2) appropriateness of cases and controls, (3) criteria to identify cases and controls, (4) standard measurement of exposure, (5) similarity in measurement of exposure for cases and controls, (6) handling of confounder, (7) strategies to handle confounder, (8) standard assessment of outcome, (9) appropriateness of duration for exposure, and (10) appropriateness of statistical analysis. Finally, seventeen cross-sectional studies and nine case–control studies were evaluated, and all received a quality score of 50% or above on the quality scale, indicating that they are low risk and included in the analysis **(S3 File).**

### Data synthesis and analysis

The data synthesis was done in a clear and detailed descriptive summary of the included studies via tabulating. The quantitative data were extracted using Microsoft Word. Heterogeneity was assessed using the standard I^2^ [26]. To minimize the random variations between the point estimates of the primary study subgroup analysis was done based on study settings (i.e., the area where studies were conducted). In addition, to identify the possible sources of heterogeneity univariate meta-regression was conducted by considering the sample size and year of publication as covariates but sample size was found to be statistically significant. Publication bias was tested by visually inspecting a funnel plot for symmetrical distribution. Furthermore, Egger’s [27] and Begg’s tests [28] (p-value < 0.05) were used to assess publication bias. Mainly, Begg’s test was used to assess studies that reported prevalence, because they usually have an asymmetric distribution and inconsistency of results is often high [28]. For pooled prevalence analysis, double arcsine-based transformed result was pooled to stabilize the variance of each study’s proportion [29]. Analyses were performed using RevMan 5.4 [30] and STATA statistical software, version 14 [31].

## Results

A total of 1,125 studies were identified from the database. Of those, 52 articles were identified from PubMed, 517 were from Medline, 40 were from Google Scholar, 502 were from Hinari, and 14 were from other sources. From these studies, 324 were excluded for being duplicates. From the remaining 801 studies, 772 articles were excluded as not being relevant after reviewing their titles and abstracts. The other 29 studies were assessed through a review of the full-text articles. Finally, 26 studies were included in the meta-analysis [17–22,32–50] (**S1 Fig**). Of the included articles, 17 were cross-sectional designs [17–22,33–42] and nine were case-control study designs [43–51] and they comprised a population of 15,637. These studies represented the following 8 regions: Amhara (n = 9), Tigray (n = 7), Southern Ethiopia (n = 4), Oromia (n = 3), Harari (n = 1), Dire-Dawa (n = 1) and Addis Ababa (n = 1). Seventeen studies were used to assess the pooled prevalence of birth asphyxia, and 22 were used to identify the risk factors associated with it. The minimum sample size from the included study was 154 [40] from Amhara, whereas the maximum sample size from the included study was 9,736 from Dire Dawa [20]. The prevalence of bith asphyxia ranged from 3.1% in Dire Dawa [20] to 41.2% in Silte Zone, Southern Ethiopia [35]. **Table 1** shows the characteristics of the studies that were included.

**Table 1:** Characteristics of included studies in the meta-analysis of the pooled prevalence and risk factors of birth asphyxia in Ethiopia.

Primary Author	Publication year	Study area	Study design	Sample size	Response rate	Prevalence (95% CI)	Study–specific factors
G/her GT et al [17]	2020	Tigray	Cross-sectional	282	94.7%	17.8(13.8, 23.0)	Pronged labor, MSAF and preeclampsia
Gebreheat G et al [38]	2018	Tigray	Cross-sectional	422	99.7%	22.1(18.4, 26.3)	Pronged labor, MSAF, caesarean delivery and LBW
Berhe YZ et al [48]	2020	Tigray	Case–control	390	100%	----	Primipara, preeclampsia, post-term pregnancy, MSAF, tight nuchal cord and non–vertex presentation.
Tasew H et al [49]	2018	Tigray	Case–control	264	100%	----	Illiteracy, LBW, preterm birth, primrara, APH and MSAF
Gebreslasie K et al [39]	2020	Tigray	Cross–sectional	648	99.8%	12.7(10.3, 15.5)	IPV, rural residence, LBW, and preterm birth
Jamie AH et al [19]	2019	Harar	Cross–sectional	258	99.2%	31.6 (26.3, 37.6)	Anaemia during pregnancy, chronic hypertension and LBW
Ibrahim A et al [20]	2017	Dire–Dawa	Cross–sectional	9,736		3.1(2.8, 3.5)	Instrumental delivery and prolonged labor
Wayessa ZJ et al [21]	2018	Oromia	Cross–sectional	371	99%	12.5 (9.5,16.3)	Complicated labor, prolonged second 2^nd^ stage labor, LBW, MSAF, tight nuchal cord, no ANC visits, non-cephalic presentation and caesarean delivery
Getachew B et al [37]	2020	Oromia	Cross–sectional	352	95	11.5 (8.5, 15.3)	Prolonged duration of labor, maternal history of khat use and LBW
Abdo RA et al [22]	2019	SNNPR	Cross–sectional	279	100%	15.1 (11.3, 19.7)	Primigravida, prolonged second stage of labor, preterm birth, MSAF and tight nuchal cord
Alemu A et al [34]	2019	SNNPR	Cross–sectional	262	97.7%	32.8 (27.4, 38.8)	Anemia during pregnancy, chronic hypertension primigravida, prolonged and LBW
Mamo SA et al [35]	2020	SNNPR	Cross–sectional	311	100%	41.2 (35.8, 46.7)	Preeclampsia, APH, GDM, PROM, Fetal distress and MSAF
Ayele MW et al [43]	2019	Amhara	Case–control	429	100%	----	APH, Maternal anemia, PROM, MSAF, Male neonatal sex, LBW and preterm birth
Gudayu TW [18]	2017	Amhara	Cross–sectional	261	100%	13.8 (10.1,18.5)	Non-vertex fetal presentation, prolonged labor MSAF, Induced and LBW
Wosenu L et al [44]	2018	Amhara	Case-control	273	98.9%	----	Prolonged labor, CD, MSAF, fetal distress and LBW
Woday A et al [32]	2019	Amhara	Cross-sectional	357	96.6%	14.8 (11.4, 18.9)	Primipara, complicated labor and PROM
Meshesha AD et al [45]	2020	Amhara	Case-control	386	100%	----	Low birth weight, born at health centers, Instrumental delivery and prolonged labor
Kibret Y et al [46]	2018	Amhara	Case-control	380	100%	----	Instrumental delivery, prolonged and complicated labour
Demisse AG et al [33]	2017	Amhara	Cross-sectional	769		12.5 (10.3, 15.0)	-----------
Mulugeta T et al [47]	2020	Addis Ababa	Case-control	213	98.59%	----	APH, LBW, preterm birth, caesarean delivery, Instrumental delivery, Fetal distress and MSAF
Selamu A et al [36]	2019	Tigray	Cross–sectional	371	100%	20(16.2, 24.3)	Induction of labor, MSAF, instrumental delivery and primipara
G/medhin M et al [50]	2020	Tigray	Case-control	662		---	APH, preeclampsia, prolonged second stage of labor, cesarean delivery, MSAF, LBW, PROM, induction and preterm birth
Asfere NW et al [40]	2018	Amhara	Cross-sectional	154	100%	29.9 (23.2, 37.5)	-----------
Bayih WA et al [41]	2020	Amhara	Cross-sectional	582	100%	28.4(24.8,32.14)	Non-cephalic presentation, PROM, MSAF, ID, caesarean delivery and prolonged labor
Lake EA et al [42]	2019	SNNPR	Cross-sectional	278	95%	25.7(20.8, 31.2)	Non-vertex presentation, prolonged labour, Primipara, LBW, MSAF, PROM, caesarean delivery, ID
Bedie NG et al [51]	2019	Oromia	Case-control	346	346	-----	Preterm, instrumental delivery, LBW, Caesarean section, PROM, Rural residence and non-vertex presentation

**Note:** APH: Antepartum hemorrhage; LBW: Low birth weight; MSAF; meconium–stained amniotic fluid, IPV: Intimate partner violence during pregnancy; PROM: Premature rupture of fetal membranes; SS: Sample size; RR: Response rate.

### Pooled prevalence of birth asphyxia

This meta-analysis indicated that the pooled prevalence of birth asphyxia in Ethiopia was found to be 19.3% (95% CI: 12.6%, 27.1%) (**S2 Fig**). This observed effect size varies somewhat from study to study. As the test statistic showed there was a significant heterogeneity among the included studies (I^2^ = 98.86%, p < 0.001) as a result a random effects meta-analysis model was used to estimate the Der Simonian and Laird’s pooled effect [52]. I^2^ (which quantifies the degree of variability between studies) for the pooled estimate can be extremely high even in the presence of modest inconsistency between studies [53]. When judging inconsistency in such situations, extent of variation in point estimates is far more important; one could even argue that the I^2^ is misleading and should not be considered. I^2^ statistic is large -but please note the I^2^ statistic is only one of several things to be considered when assessing heterogeneity. A funnel plot created to investigate publication bias showed an asymmetrical distribution (**S3 Fig**). The results of Begg’s tests also indicated statistically significant publication bias (P = 0.04). The possible sources of heterogeneity were explored using Univariate meta-regression using publication year and sample size as covariates. Sample size was statistically significant for explaining heterogeneity (**Table 2**). As subgroup analysis indicated, the pooled prevalence of birth asphyxia varied widely across the geographic areas, ranging from 28.2% in Southern Ethiopia to 3.4% in other areas **(Table 3).** No studies were found to be outside the confidence bounds in sensitive analysis, implying that all studies had a nearly equal influence on the pooled prevalence **(S4 Fig)**.

**Table 2:** Related factors with heterogeneity of birth asphyxia prevalence in this meta-analysis, in Ethiopia, 2021.

Variation	Coefficient	P-value
Publication year	3.4(-.7.0,7.5)	0.10
Sample size	-.002(-0.004,-0.001)	0.00

**Table 3:** Subgroup analysis of pooled prevalence of birth asphyxia in Ethiopia, 2020.

Variables	Characteristics	Included studies	Number of respondents	Estimated prevalence (95%CI)
By region	SNNPR	4	1,110	28.2(17.5,40.1)
	Oromia	2	846	12.0(9.7,14.5)
	Amhara	5	2,111	19.2(12.3, 27.2)
	Tigray	4	1706	18.0(13.5,22.8)
	Dire-Dawa and Harari	2	9994	3.4(3.1,3.7)

### Risk factors associated with birth asphyxia in Ethiopia

#### Antepartum hemorrhage (APH)

The likelihood of birth asphyxia occurrence was 4.7 times higher among mothers who experienced APH than those who did not (OR: 4.7; 95% CI: 3.5, 6.1**) (S5 Fig)**. No heterogeneity was observed among the included studies; hence, a fixed effects model was used in the analysis. The results of Egger’s and Begg’s tests indicated no statistically significant publication bias (P = 0.76 and P = 0.44, respectively).

#### Premature rupture of fetal membranes (PROM)

The pooled odds ratio indicated that PROM was positively associated with birth asphyxia (OR: 3.96; 95% CI: 12.37, 6.59) **(S6 Fig**). High heterogeneity (I^2^ = 87.0% and p = 0.0001) was observed among the included studies; hence, a random effects model was used in the analysis. The results of Egger’s and Begg’s tests indicated no statistically significant publication bias (P = 0.9 and P = 0.6, respectively).

*Parity*. The pooled effect of six studies showed that primiparas were 2.84 times more likely to develop birth asphyxia than multipara (OR: 2.84; 95% CI: 1.95, 4.13). A heterogeneity test indicated an I^2^ of 62%. Due to the observed variability, a random effects model was used in the analysis **(S7 Fig).** The results of Egger’s and Begg’s tests indicated no statistically significant publication bias (P = 1.0 and P = 0.7, respectively).

#### Birth weight

LBW was revealed a 5.6 times higher risk of birth asphyxia than normal birth weight (OR = 5.6; 95% CI: 4.7, 6.7) **(S8 Fig)**. No heterogeneity was observed among the included studies; hence random effect analysis model was used to examine the association between birth asphyxia and LBW. The results of Egger’s and Begg’s tests indicated no statistically significant publication bias (P = 1.0 and P = 0.7, respectively).

#### Meconium stained amniotic fluid

Women who experienced intrauterine meconium release were 5.6 times more likely to experience birth asphyxia than those who did not (OR: 5.6; 95% CI: 4.12, 7.50). Moderate heterogeneity (I^2^ = 69%; P < 0.0001) was observed across the studies; hence, a random effects model was employed to estimate the pooled effect **(S9 Fig)**. The results of Egger’s and Begg’s tests indicated no statistically significant publication bias (P = 0.02 and P = 0.03, respectively).

#### Prolonged labor

Birth asphyxia was 4.2 times more likely to occur in women who experienced prolonged labour than in those who did not (OR: 4.2; 95% CI: 2.67, 6.6). High heterogeneity (I^2^ = 83.0%; p< 0.0001) was observed across the studies; hence, a random effects model was employed to estimate the pooled effect **(S10 Fig)**. The results of Egger’s and Begg’s tests indicated no statistically significant publication bias (P = 0.5 and P = 0.9, respectively).

#### Abnormal fetal presentation

Abnormal presentation was associated with a risk of birth asphyxia 5.65 times higher than normal (vertex) presentation (OR = 5.6; 95% CI: 3.84, 8.30). No heterogeneity was observed (I^2^ = 62%; p = 0.007) among the included studies; therefore, a fixed effects model was used in the analysis **(S11 Fig)**. The results of Egger’s and Begg’s tests indicated no statistically significant publication bias (P = 0.2 and P = 0.2, respectively).

#### Maternal anaemia

Maternal anaemia was found to be a significant risk factor for birth asphyxia (OR: 5.1; 95% CI: 2.6, 9.9). Moderate heterogeneity (I^2^ = 69%; P = 0.02) was observed across the studies; therefore, a random effects model was used to estimate the pooled effect **(S12 Fig**). The results of Egger’s and Begg’s tests indicated no statistically significant publication bias (P = 1.0 and P = 0.9, respectively).

#### Chronic hypertension

The probability of developing birth asphyxia among women who had chronic hypertension was 2.5 times higher than those who did not (OR = 2.52; 95% CI: 1.69, 3.752). No heterogeneity was observed; therefore, a fixed effects model was used to estimate the pooled effect **(S13 Fig).** The results of Egger’s and Begg’s tests indicated no statistically significant publication bias (P = 1.0 and P = 0.15, respectively).

#### Preterm birth

The risk of developing birth asphyxia was 4.1 times higher in women who had PTB than in those who did not (OR = 4.1; 95% CI: 2.9, 5.8). High heterogeneity (I^2^ = 60%; P = 0.007) was observed across the studies; therefore, a random effects model was employed to estimate the pooled effect (**S13 Fig**). The results of Egger’s and Begg’s tests indicated no statistically significant publication bias (P = 0.5 and P = 0.8, respectively).

#### Place of residence

This meta-analysis found that birth asphyxia was associated with rural residence (OR: 2.6; 95% CI: 2.00, 3.58). No heterogeneity was observed among the included studies; hence, a fixed effects model was used in the analysis **(S15 Fig).** The results of Egger’s and Begg’s tests indicated no statistically significant publication bias (P = 1.0 and P = 0.5, respectively).

#### Caesarean delivery

This meta-analysis also found that birth asphyxia was 4.4 times more likely to occur in women delivering by caesarean section than in those delivering by normal birth (OR = 4.4; 95% CI, 3.1, 6.2). A heterogeneity test indicated an I^2^ of 61%. Due to the observed variability, a random effects model was used in the analysis **(S16 Fig).** The results of Egger’s and Begg’s tests indicated no statistically significant publication bias (P = 0.4 and P = 0.6, respectively).

#### Operative vaginal/Instrumental delivery

Women who underwent operative vaginal delivery were nearly six times more likely to develop birth asphyxia than those who did not (OR: 4.9; 95% CI: 3.54, 6.7). As heterogeneity was observed among the included studies (I^2^ = 31%; P = 0.17), a random effects model was used in the analysis **(S17 Fig).** The results of Egger’s and Begg’s tests indicated no statistically significant publication bias (P = 0.2 and P = 0.7, respectively).

#### Preeclampsia

This meta-analysis found that neonates born to mothers who had preeclampsia were 3.9 times more likely to develop birth asphyxia than those those who did not (OR = 3.9; 95% CI: 2.1, 7.4). High heterogeneity (I^2^ = 80%; P = 0.0006) was observed among the included studies; hence, a random effects model was used in the analysis **(S18 Fig)**. The results of Egger’s and Begg’s tests indicated no statistically significant publication bias (P = 0.7 and P = 0.8, respectively).

#### Complications during labor

This meta-analysis found that birth asphyxia was associated with complicated labor (OR: 3.7; 95% CI: 2.8, 4.9). No heterogeneity [I^2^ = 0%; p = 0.46] was observed among the included studies; hence, a fixed effects model was used in the analysis **(S19 Fig)**. The results of Egger’s and Begg’s tests indicated no statistically significant publication bias (P = 0.7 and P = 0.4, respectively).

#### Induction of labor

Women who underwent induction were 3.8 times more likely to develop birth asphyxia than those who did not (OR: 3.9; 95% CI: 2.6, 5.6) (**S20 Fig**). Moderate heterogeneity (I^2^ = 12%; P = 0.02) was observed; therefore, a random effects model was used to estimate the pooled effect. The results of Egger’s and Begg’s tests indicated no statistically significant publication bias (P = 0.7 and P = 0.2, respectively).

#### Prolonged second stage of labor

Birth asphyxia was more or three times more likely to occur in women who experienced prolonged second stage of labor than in those who did not (OR: 3.2; 95% CI: 2.3, 4.5). No heterogeneity was observed among the included studies; hence, a fixed effects model was used in the analysis **(S21 Fig).** The results of Egger’s and Begg’s tests indicated no statistically significant publication bias (P = 1.0 and P = 0.1, respectively).

#### Tight nuchal cord

This meta-analysis found that birth asphyxia was associated with tight nuchal cord (OR: 3.4; 95% CI: 2.09, 5.62). Moderate heterogeneity [I^2^ = 39%; p = 0.16] was observed among the included studies; hence, a fixed effects model was used in the analysis (**S22 Fig**). The results of Egger’s and Begg’s tests indicated no statistically significant publication bias (P = 0.1 and P = 0.9, respectively).

#### Maternal education

This review also indicated significant associa​tion between mothers’ educational status and birth asphyxia. Mothers who can read and write were 4.2 times more likely to give asphyxiated baby as compare to who can’t read and write(OR = 4.2;95% CI:1.7,10.6). Heterogeneity test indicated I^2^ = 0.0%, hence fixed effect model was assumed in the analysis **(S23 Fig).**

#### Fetal distress

In this meta-analysis, fetal distress was significantly associated with higher odds of birth asphyxia (OR = 3.4; 95% CI: 2.4, 4.8). High heterogeneity (I^2^ = 88%; P <0.01) was observed; therefore, a random effects model was used to estimate the pooled effect **(S24 Fig).** The results of Egger’s and Begg’s tests indicated no statistically significant publication bias (P = 1.0 and P = 0.4, respectively).

#### Antenatal care follow up (ANC)

Mothers who didn’t attend ANC were nearly three times more likely to have asphyxiated newborn when compared to those who attended antenatal care follow up (OR = 2.94; 95% CI: 2.1, 4.1). Heterogeneity (I^2^ = 12.0%) was observed among the included studies; hence, a random effects model was used in the analyses **(S25 Fig)**. The results of Egger’s and Begg’s tests indicated no statistically significant publication bias (P = 0.73 and P = 0.24, respectively).

#### Post term birth

Neonate who was born with gestational age of > 42 week was 3.16 time more likely develop birth asphyxia as compared to term (OR = 3.2; 95% CI:1.60, 6.26). No heterogeneity was observed among the included studies; hence, a fixed effects model was used in the analyses **(S26 Fig)**. The results of Egger’s and Begg’s tests indicated no statistically significant publication bias (P = 0.73 and P = 0.24, respectively).

## Discussion

This systematic review and meta-analysis was performed to estimate the pooled prevalence of birth asphyxia in Ethiopia and, further, to explore the factors that contribute to it. According to this meta-analysis, Ethiopia’s pooled prevalence of birth asphyxia was found to be 19.3%. Yet this finding is higher than the percentages reported in primary studies conducted in Vietnam (2%), India (6.6%), Tanzania (11.5%) and Nigeria (12.8%) [54–57]. As well, a higher prevalence was observed in this study, compared to other meta-analysis in East and Central Africa (16.5%) [58].

However, the pooled prevalence of birth asphyxia was lower, compared to those in other countries, such as Kenya, Pakstan, Nigeria, and Zambia, reported as 29.1%, 56.9%, 30.1%, and 23%, respectively [59–62]. This variation may be attributed to the differences in terms of study setting, design, population and the manner in which awareness is created with regard to adverse birth outcomes for the general community and difference in community engagement in Ethiopia’s maternal health issue. Another possible reason could be the differences in the implementation of services for mothers and their new-born babies.

As per the findings of the subgroup analysis, the pooled prevalence of birth asphyxia varied widely across the geographic areas ranges from 28.6% in Southern Ethiopia to 3.1% in Dire-Dawa. This variation could possibly be ascribed to the cultural variation across the country’s regions, the number of studies included and disparities in educational status.

Meta-analysis was used to examine the association between birth asphyxia and antepartum haemorrhage in the context of Ethiopia. Accordingly, antepartum haemorrhage was found to be significantly associated with the prevalence of birth asphyxia. This finding is consistent with studies conducted in India, Colombia, Indonesia and Pakistan [53–65]. A plausible justification for this could be that bleeding during pregnancy reduces uteroplacental perfusion, thus resulting in birth asphyxia.

The findings of this meta-analysis indicated that premature rupture of fetal membranes was positively associated with birth asphyxia, which is consistent with the results of a study conducted in India, Cameroon and Pakistan and Bangladesh [55,66–68]. A probable explanation is that after a premature rupture of membranes, the umbilical cord is no longer cushioned by the amniotic fluid, and can become compressed. Cord compression is dangerous because the flow of oxygen-rich blood to the baby is interrupted, and the baby may experience birth asphyxia [69]. Another justification may be that without the protection of the amniotic sac, maternal infections can be easily transmitted to the baby, potential leading to sepsis and brain damage, which may result in birth asphyxia [70].

In this meta-analysis, primiparas were more likely to develop birth asphyxia than multipara. This finding is consistent with previous studies conducted in Pakistan [60,67,71], Indonesia [72], India [55] and Sweden [73]. These findings could be attributed to the fact that primiparas do not usually receive care during pregnancy, they are less interested in the demands of the pregnancy and often neglect maternal care services [74]. Low-birth-weight babies were found to be at greater risk of developing birth asphyxia than babies with a normal birth weight, a finding which is in line with studies carried out in Nigeria [61], Pakistan [60,65,75], Zambia [62] and Indonesia [64,75]. A possible reason for this finding may be due to the fact that birth asphyxia occurs because of a lack of pulmonary surfactant, with the underdevelopment of the lungs, added to the fact that the respiratory muscles are still weak and the ribs can easily become curved [76,77].

The result of this review reveals that the release of intrauterine meconium increases the likelihood of birth asphyxia. This finding is again supported by studies conducted in in Pakistan [56,62], Indonesia [72] and India [78,79]. A possible reason for this finding is that the presence of meconium in amniotic fluid enhances the chance of meconium aspiration occurring during intrauterine gasping or during the initial breaths taken after birth, which may cause acute airway obstruction, surfactant dysfunction or inactivation [80]. Birth asphyxia is more likely to occur in women who experience prolonged labor than in women who do not, as has previously been reported in Nigeria [61,81,82] and Pakistan [65,67]. The probable reason for this is the fact that women who undergo prolonged labor have more negative birth experiences than women who undergo normal labour [83]. Moreover, birth asphyxia is likely to stem from babies who are born as a result of prolonged labour experiencing the stress of too many uterine contractions or umbilical cord problems [83,84].

This meta-analysis revealed non-vertex presentation to be significantly associated with birth asphyxia, which is in accordance with the findings of prior studies conducted in Cameroon [64], Pakistan [65,68] and India [79]. One probable explanation for this findings is that abnormal presentations (e.g. breach, brow, face, etc.) increase the risk of numerous complications, including umbilical cord prolapse or compression, which can result in birth asphyxia [85]. Another possible explanation is that abnormal presentations are mismanaged, putting the baby at risk of a brain bleed, which could lead to fetal hypoxia [86]. In the present meta-analysis, maternal anaemia was found to be a significant risk factor for birth asphyxia. This might be due to the fact that lower maternal haemoglobin concentrations during labor are associated with lower oxygen perfusion to the foetus and, therefore, with a higher risk of birth asphyxia. This finding is supported by the findings of studies conducted in Pakistan [67], India [79] and Indonesia [87].

The results of this meta-analysis also revealed that the risk of developing birth asphyxia is higher in relation to preterm birth than full-term birth. This finding is supported by studies conducted in Pakistan [65,71], Zambia [62], Indonesia [64,75] and India [79], which all reported that premature babies were more likely to develop birth asphyxia than full-term babies. The reason for this could be that a premature baby’s organs are not yet fully mature, which means that the respiratory system, especially the baby’s lungs, cannot work optimally [88]. Another reason could be related to the inadequate surfactant noted in premature babies, which can result in problems concerning lung development [89]. Additionally, a premature baby’s respiratory muscles are likely to be weak, resulting in the sound of the baby’s crying also being weak, which can cause the baby to experience asphyxia [88,89].

Interestingly, this meta-analysis suggested residing in a rural area to be significantly associated with birth asphyxia. This finding might be explained by the common characteristics of the rural populations, namely the lower proportion of educated mothers, the lower degree of knowledge concerning adverse birth outcomes, the lack of nearby health-care services and the lower access to information when compared with urban populations [90]. Due to these characteristics, rural women are more likely to be both unaware of potential adverse birth outcomes and less likely to receive adequate care during labour, which results in an increased likelihood of experiencing adverse birth outcome such as birth asphyxia [90,91].

According to this meta-analysis, birth asphyxia is associated with birth via caesarean section. A possible reason for this finding is the fact that during vaginal delivery, approximately a third of the foetal lung fluid is removed by squeezing the baby’s chest, which does not occur during delivery by means of caesarean section. In addition, less surfactant is secreted to the alveolar surface during caesarean section birth, which gives rise to a higher risk of developing birth asphyxia [92,93]. Similarly, studies conducted in Nigeria, Indonesia and Pakistan have found caesarean delivery to be significantly associated with birth asphyxia [61,64,65]. The likelihood of developing birth asphyxia is 2.52 times higher among women who experience chronic hypertension than among women who do not. This finding is nearly universal, with pregnancies complicated by chronic hypertension being reported to result in adverse outcomes such as low birth weight, preterm birth, perinatal death and birth asphyxia [73,94].

The present meta-analysis also revealed that the babies of women who undergo operative vaginal delivery are nearly six times more likely to develop birth asphyxia than the babies of women who do not. This finding is in accordance with the findings of studies conducted in Indonesia [72] and India [78]. The likely reason behind this finding is the fact that the use of forceps and/or vacuum extractors can result in brain bleeds [95,96].

Moreover, this meta-analysis found preeclampsia to be associated with birth asphyxia, which is consistent with the findings of studies conducted in Nigeria [61], Cameroon [66], Pakistan [69], Indonesia [64,72,75] and India [79]. One probable explanation for this is that preeclampsia is a progressive disorder and, in most cases, labour needs to be induced at around the 34^th^ to 38^th^ week of pregnancy to safeguard the health of the mother and the foetus [97]. However, the necessity of preterm birth often has adverse consequences in terms of important neonatal outcomes, including birth asphyxia [98]. Another explanation could be that preeclampsia can result in blood pressure issues in the mother, including hypertensive crisis. This can greatly decrease the supply of oxygen-rich blood available to the baby. If not properly controlled, this condition can be fatal to an unborn child [94,98].

This meta-analysis found that birth asphyxia was associated with tight nuchal cord. This is comparable with study done in Pakistan [65]. A possible explanation is that tight nuchal cord constrict umbilical vessels and consequent hypoxic result [99,100].

In this meta-analysis, fetal distress was significantly associated with higher odds of birth asphyxia. This finding is consistent with the studies done in Pakistan, Indonesia and India [73,75,82]. The possible explanation is that fetal distress mainly results from insufficient placental perfusion or any factor during labor that will impair fetal oxygenation, which can cause further difficulty initiating and sustaining breathing after birth, ending up with asphyxia [101]. Post term delivery was significantly associated with an increased odds of birth asphyxia. This finding is consistent to the study done in Indonesia [64]. Post term pregnancy is an important obstetric issue because of the increased risk of placental insufficiency, oligohydramnios and meconium aspiration which will result in intrauterine asphyxia and may born in a birth asphyxia condition [84,94].

Mothers who did not attend four or more antenatal care visits increased the likelihood of birth asphyxia when compared to those who attended ANC follow up. This finding is consistent with primary studies conducted in Pakistan [60], India [75,78] and Nigeria [61,83]. This could be due to the fact that mothers who had ANC visits may have adequate awareness about the danger signs related to pregnancy, labor and the postnatal period, as well as other health-related education from health professionals during their follow up visits. This might reduce the delay in getting appropriate care during labor, which is more likely to decrease adverse birth outcomes including birth asphyxia.

Women who underwent induction were more likely to develop birth asphyxia than those who did not. This finding was supported by a primary studies in a remote India [78,79]. The possible reason for this finding might be due to the fact that induction drugs can cause excess uterine contractions (hyper stimulation), which may lead to fetal heart rate changes [102]. When contractions are too fast and strong, the placenta, which helps carry oxygen-rich blood to the baby, often cannot recharge with an adequate supply of this blood for the baby [102,103]. Sometimes intense contractions caused by labor induction drugs can cause head trauma [102–104].

Prolonged second stage labor is associated with an increased risk of birth asphyxia, like that reported in India, Colombia, Pakistan and Sweden [55,63,67,105]. This may be due to that second stage of labor is last part of labor and characterised by high uterine contraction and involves period of relative lack of oxygen, as the infant’s head and the umbilical cord are compressed by contractions in the birth canal so in the prolonged second stage of labor increase the above condition persist and resulting in birth asphyxia [106,107]. The educational status of women was found to be one of the factors in this review: i.e. women who could not read and write were more likely to develop birth asphyxia when compared to women who had attended secondary education and above. This finding is consistent to that of a study conducted in Pakistan [65]. This might be because educated women are more aware of maternal health services and have more autonomy and freedom to make decisions about their own health, which eventually enhances their health-seeking behaviour.

Intermittent auscultation with fetal fetoschope has long been the primary method of fetal monitoring in low-resource settings, yet more than half of newborns who develop birth asphyxia have a normal fetal heart rate during labor. As a result, developing countries should increase their use of continuous fetal heart rate (FHR) monitoring, which has aided in the faster detection of an aberrant FHR and may be a breakthrough in detecting foetuses at high risk of birth asphyxia [108]. Reduced birth asphyxia has been linked to regular helping baby’s breath (HBB) training for health workers, improved communication, and increased availability of resuscitation resources and training of delivery personnel in neonatal resuscitation [108,109]. Fetal asphyxia is best treated as a team effort involving obstetrics, paediatrics, and anaesthesia [110].

Similar to other meta-analyses, the present meta-analysis had a number of limitations. First, only articles and reports published in English were considered in this review, which sought to investigate birth asphyxia in the Ethiopian context. In addition, the majority of studies included in the review were cross-sectional in nature, which limited our ability to assess cause–effect relationships and might have resulted in the outcome variable being affected by other confounding variables. Furthermore, this meta-analysis only included studies conducted in seven regions of Ethiopia, which means that some regions may be under-represented due to the limited number of studies that were included. Additionally, some variables associated with birth asphyxia that were reported in different primary articles conducted in Ethiopia were not included in this meta-analysis because they were reported in only one article and/or classified in a different way in the included articles. Another limitation of this study is the inability to use certain databases for different reasons. Finally, in all studies conducted in Ethiopia and the included study, the Apgar score was used to identify asphyxia. However, using the Apgar score alone to identify birth asphyxia has been found to be inappropriate as it is an expression of the infant’s physiological condition at one point in time, which includes subjective components.

## Conclusion

According to the findings of this study, birth asphyxia is an unresolved public health problem in the Ethiopia. It also revealed that antepartum haemorrhage, premature rapture of fetal membranes residing in a rural area, primipara, meconium-stained amniotic fluid, prolonged labour, abnormal foetal presentation, preterm and post-term birth, caesarean and operative vaginal delivery, preeclampsia, tight nuchal cord, maternal anaemia, induction of labour, antenatal visits, prolonged second-stage labour and chronic hypertension are all significantly associated with birth asphyxia. Therefore, the concerned body needs to pay attention to the above risk factors in order to decrease the country’s birth asphyxia. The results of this review and meta-analysis could help policymakers and medical professionals to design appropriate national interventions to reduce/alleviate birth asphyxia. All primary studies conducted in Ethiopia have not included data on umbilical cord gas, neurological exam, information on therapeutic hypothermia or neurodevelopmental follow-up of asphyxiated newborns, so the researchers should consider these areas.

## Supporting information

S1 Fig Diagram of information flow through phases of systematic review. (TIF)

S2 Fig Forest plot of the pooled prevalence of birth asphyxia in Ethiopia. (TIF)

S3 Fig Funnel plot for publication bias, logprevalence represented in the X-Axis and standard error of logprevalence in the Y-Axis. (TIF)

S4 Fig The sensitivity analysis showed the pooled prevalence when the studies omitted step by step. (TIF)

S5 Fig Association of APH with birth asphyxia in Ethiopia. (TIF)

S6 Fig The pooled odds ratio of the association between PROM and birth asphyxia in Ethiopia. (TIF)

S7 Fig The pooled odds ratio of the association between primipara and birth asphyxia in Ethiopia. (TIF)

S8 Fig The pooled odds ratio of the association between LBW and birth asphyxia in Ethiopia. (TIF)

S9 Fig The pooled odds ratio of the association between meconium stained amniotic fluid and birth asphyxia in Ethiopia. (TIF)

S10 Fig The pooled odds ratio of the association between prolonged labor and birth asphyxia in Ethiopia. (TIF)

S11 Fig The pooled odds ratio of the association between abnormal fetal presentation and birth asphyxia in Ethiopia. (TIF)

S12 Fig The pooled odds ratio of the association between maternal anemia and birth asphyxia in Ethiopia. (TIF)

S13 Fig The pooled odds ratio of the association between chronic hypertension and birth asphyxia in Ethiopia. (TIF)

S14 Fig The pooled odds ratio of the association between preterm and birth asphyxia in Ethiopia. (TIF)

S15 Fig Association of place of residence with birth asphyxia in Ethiopia. (TIF)

S16 Fig The pooled odds ratio of the association caesarean delivery and birth asphyxia in Ethiopia. (TIF)

S17 Fig The pooled odds ratio of the association operative vaginal and birth asphyxia in Ethiopia. (TIF)

S18 Fig The pooled odds ratio of the association preeclampsia and birth asphyxia in Ethiopia. (TIF)

S19 Fig The pooled odds ratio of the association complications during labor and birth asphyxia in Ethiopia. (TIF)

S20 Fig The pooled odds ratio of the association induction and birth asphyxia in Ethiopia. (TIF)

S21 Fig The pooled odds ratio of the association prolonged second stage of labor and birth asphyxia in Ethiopia. (TIF)

S22 Fig The pooled odds ratio of the association tight nuchal cord and birth asphyxia in Ethiopia. (TIF)

S23 Fig The pooled odds ratio of the association maternal education and birth asphyxia in Ethiopia. (TIF)

S24 Fig The pooled odds ratio of the association fetal distress and birth asphyxia in Ethiopia. (TIF)

S25 Fig The pooled odds ratio of the association ANC and birth asphyxia in Ethiopia. (TIF)

S26 Fig The pooled odds ratio of the association post term and birth asphyxia in Ethiopia. (TIF)

S1 File Preferred reporting items for systematic review and meta-analysis protocols; recommended items to address in a systematic review and meta-analysis protocol. (DOCX)

S2 File Search terms summary. (DOCX)

S3 File Quality appraisal of included studies. (DOCX)

## Abbreviations

ANC: Antenatal Care
APH: Antepartum hemorrhage
EDHS: Ethiopian Demographic and Health Survey
HSDP: Health Sector Development Plan
OR: Odds Ratio
PROM: Premature rupture of fetal membrane
WHO: World Health Organization
