# Supplementary figures and images for: Prevalence and risk factors associated with birth asphyxia among neonates delivered in Ethiopia: A systematic review and meta-analysis
Source: PLoS One. 2021 Aug 5;16(8):e0255488. doi: 10.1371/journal.pone.0255488 (PMC8341515; doi:10.1371/journal.pone.0255488)

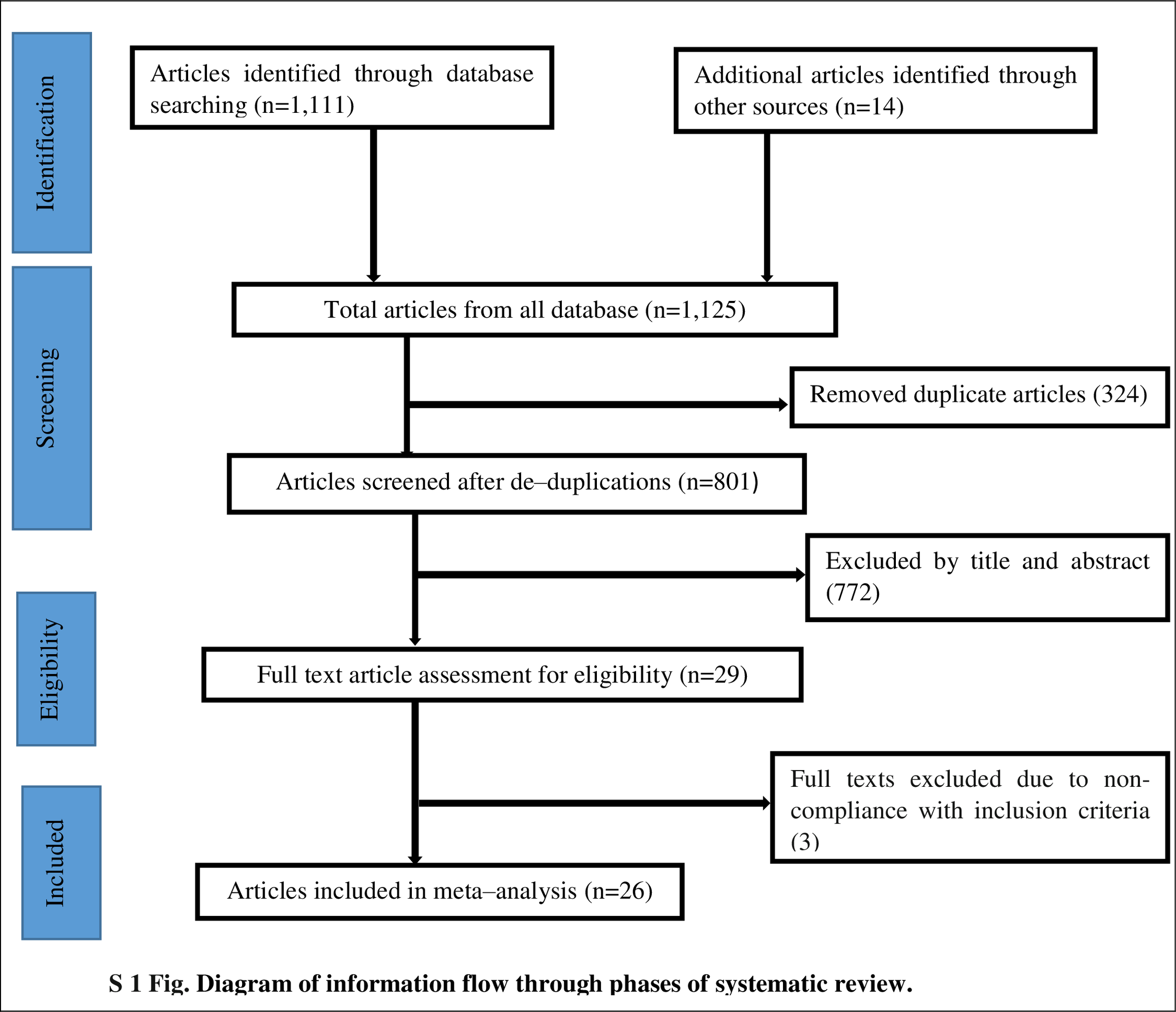

Supplement: S1 Fig — (TIF) [file pone.0255488.s001.tif]

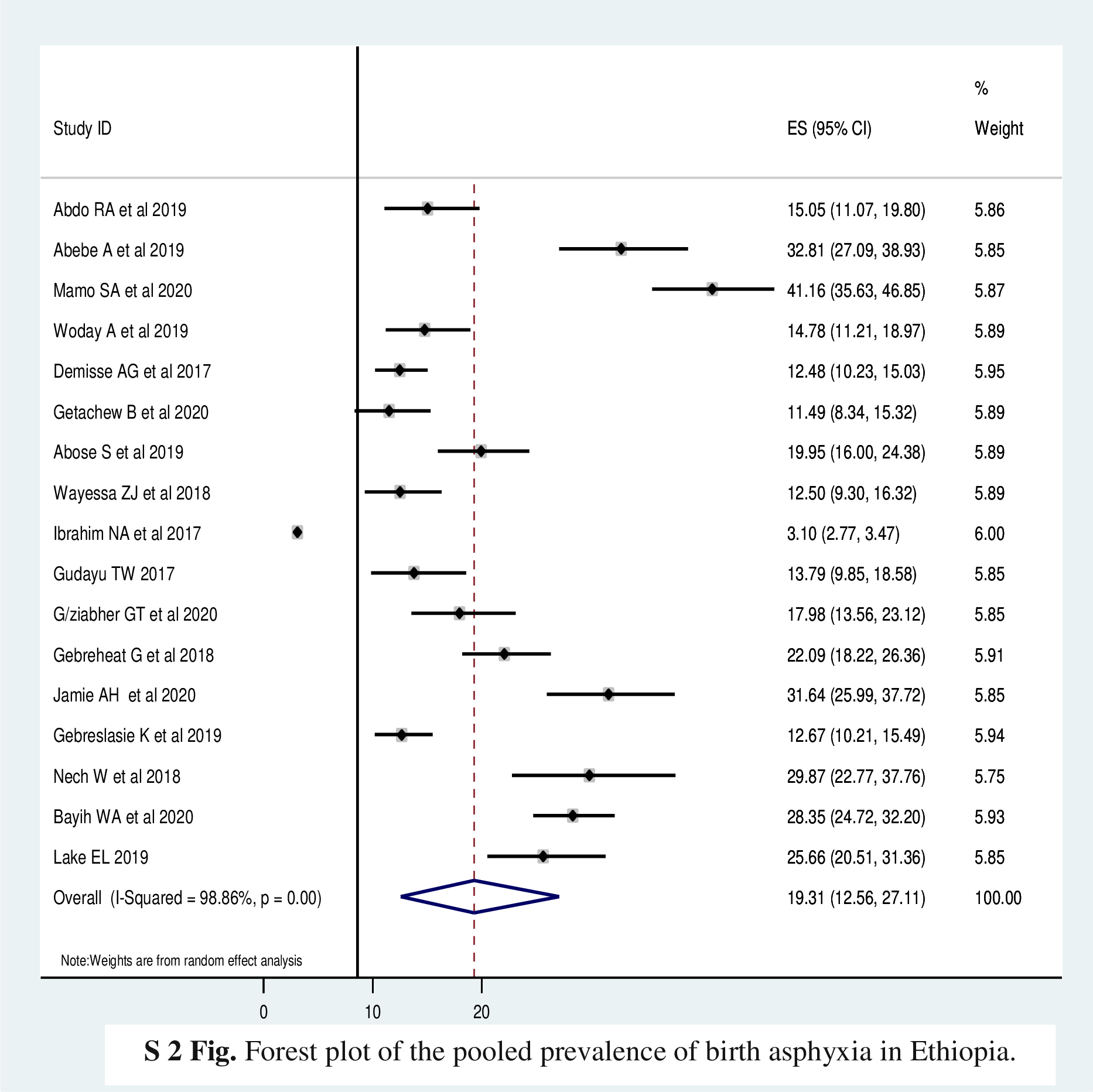

Supplement: S2 Fig — (TIF) [file pone.0255488.s002.tif]

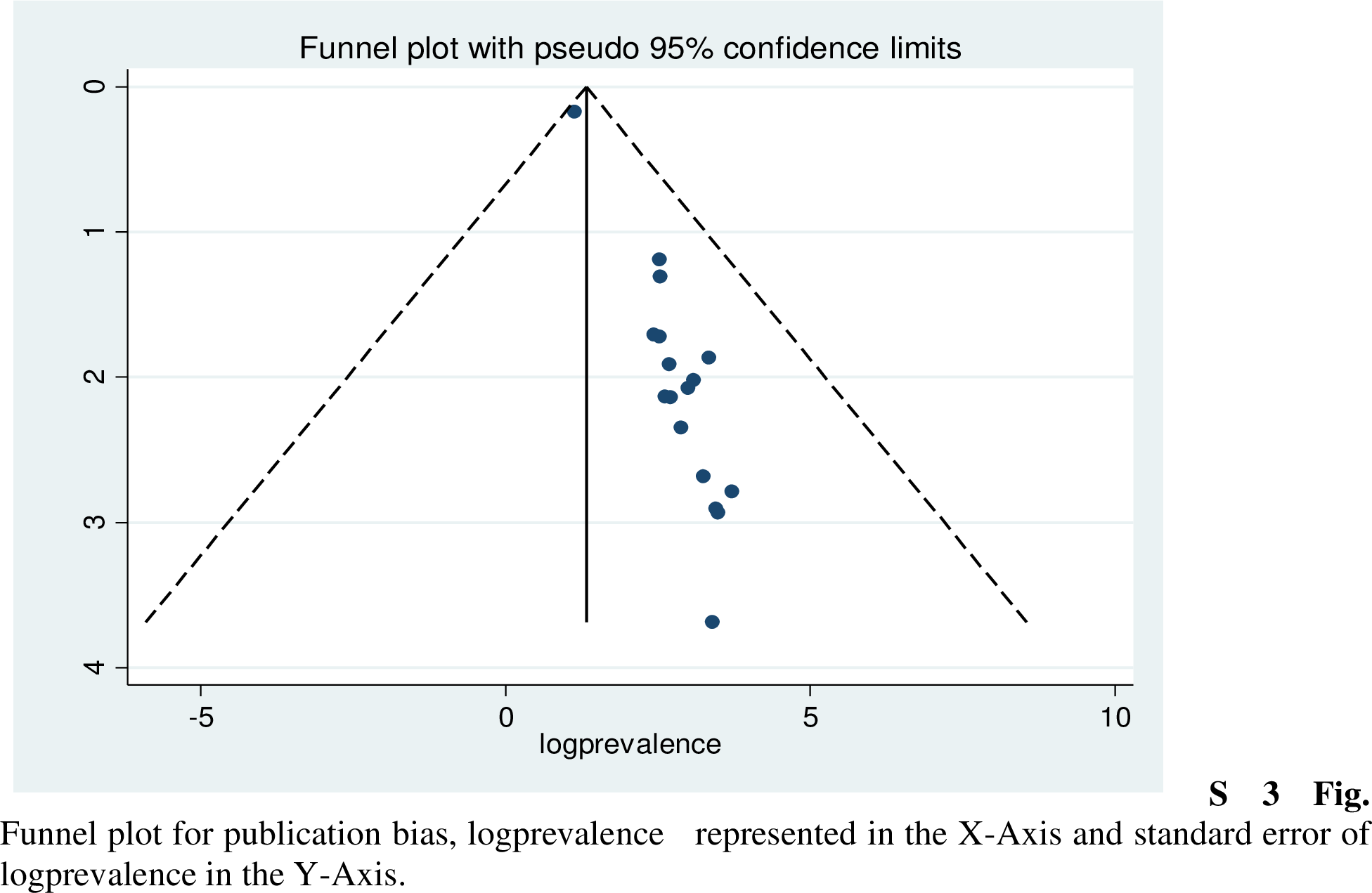

Supplement: S3 Fig — (TIF) [file pone.0255488.s003.tif]

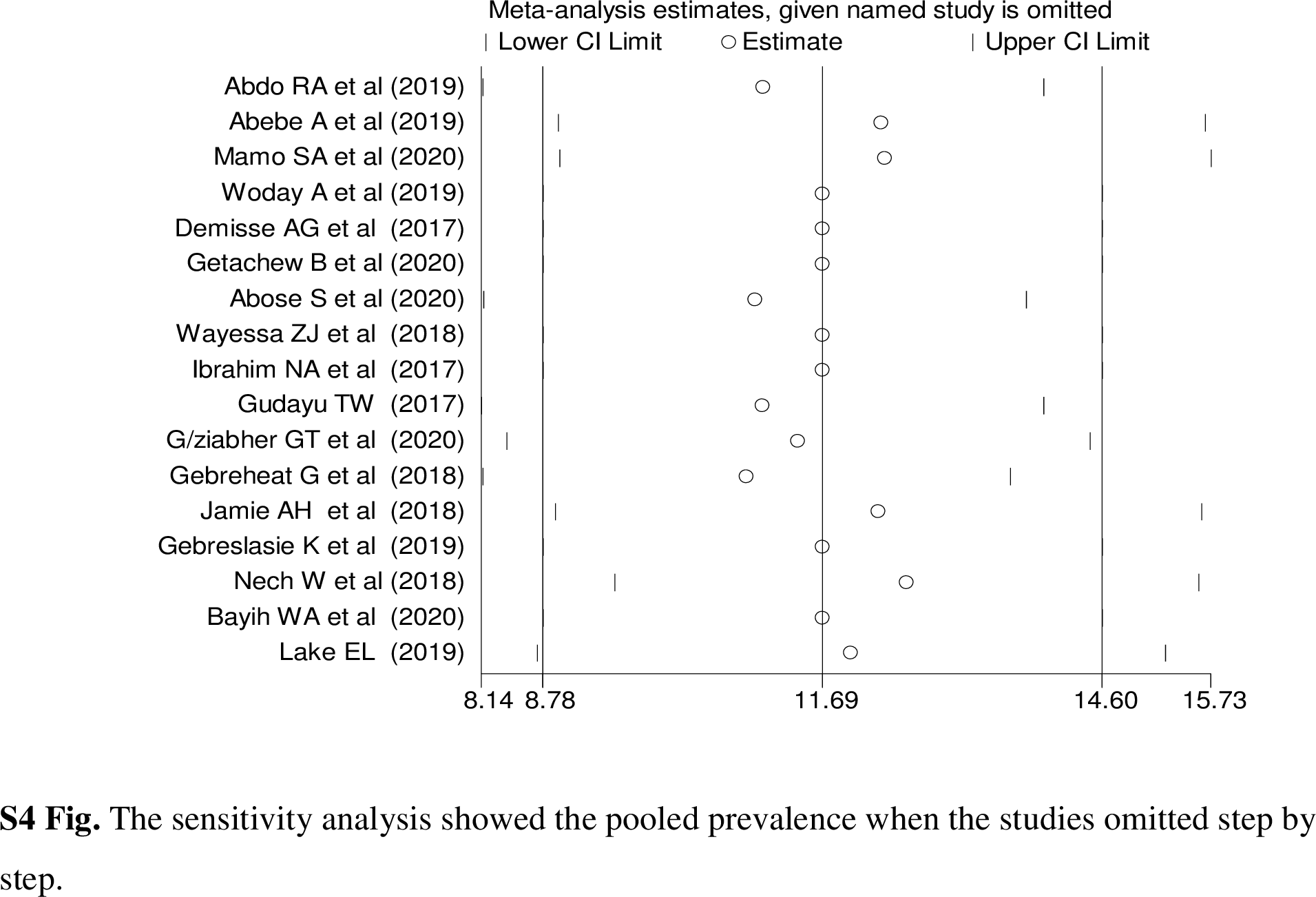

Supplement: S4 Fig — (TIF) [file pone.0255488.s004.tif]

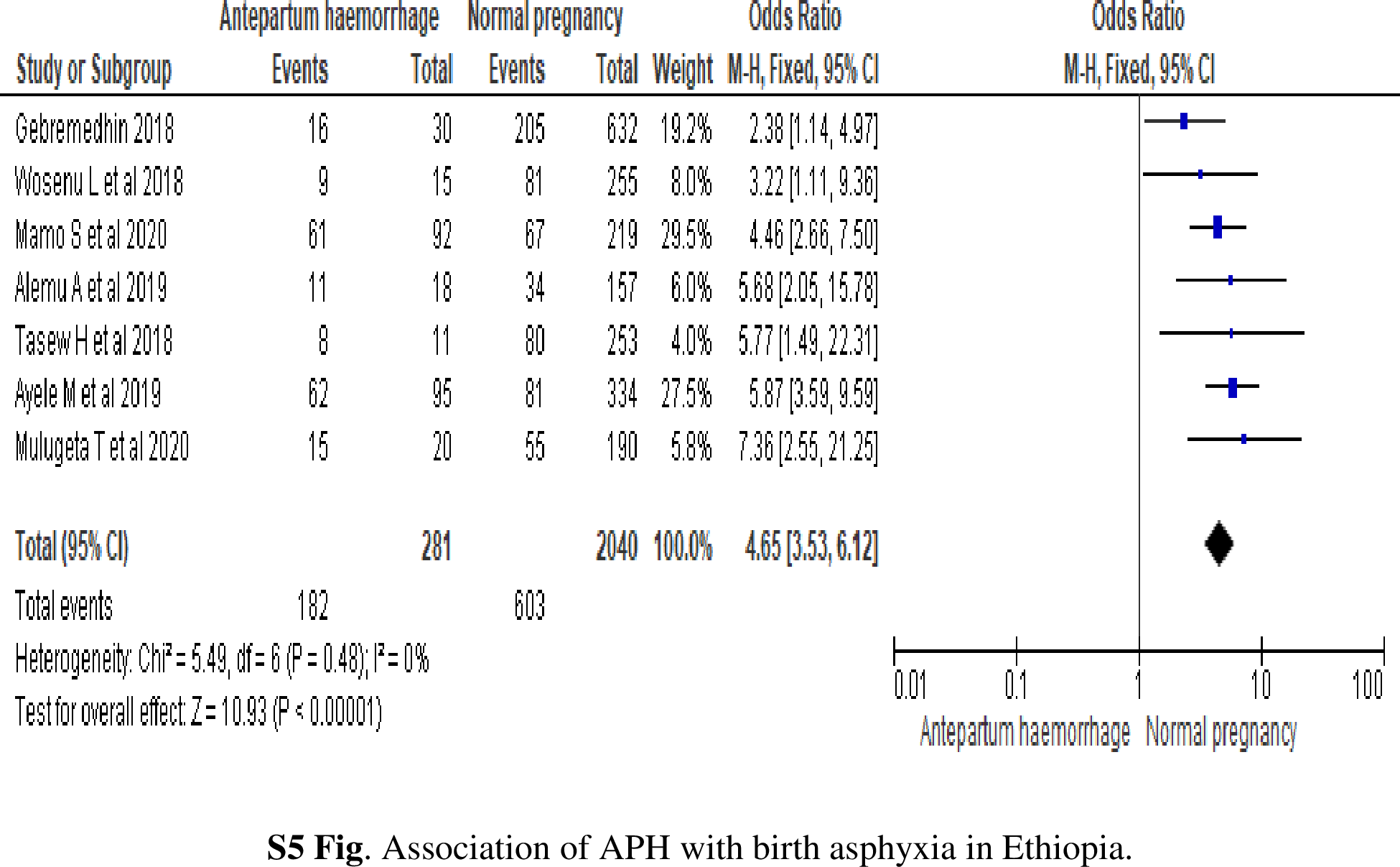

Supplement: S5 Fig — (TIF) [file pone.0255488.s005.tif]

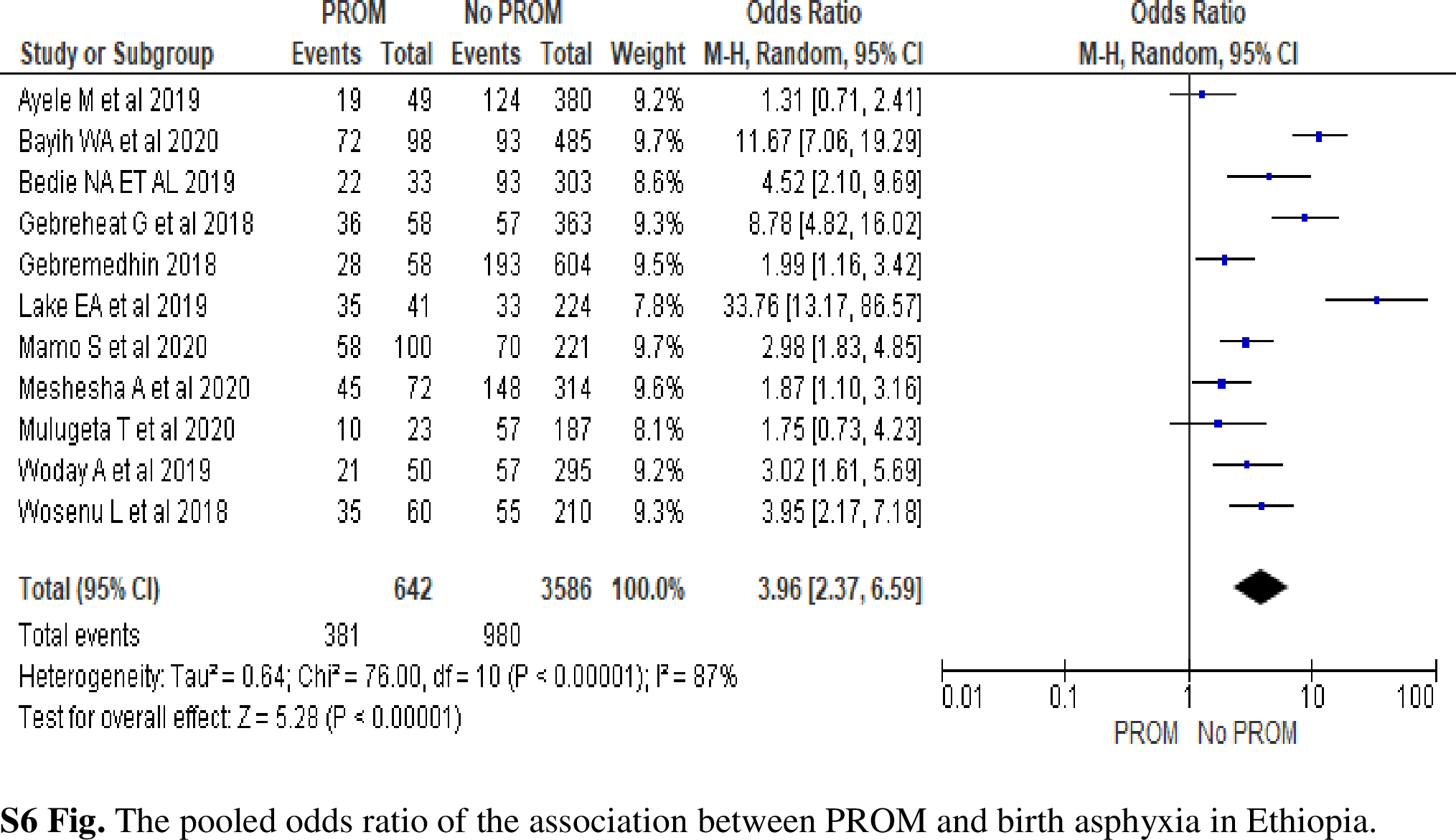

Supplement: S6 Fig — (TIF) [file pone.0255488.s006.tif]

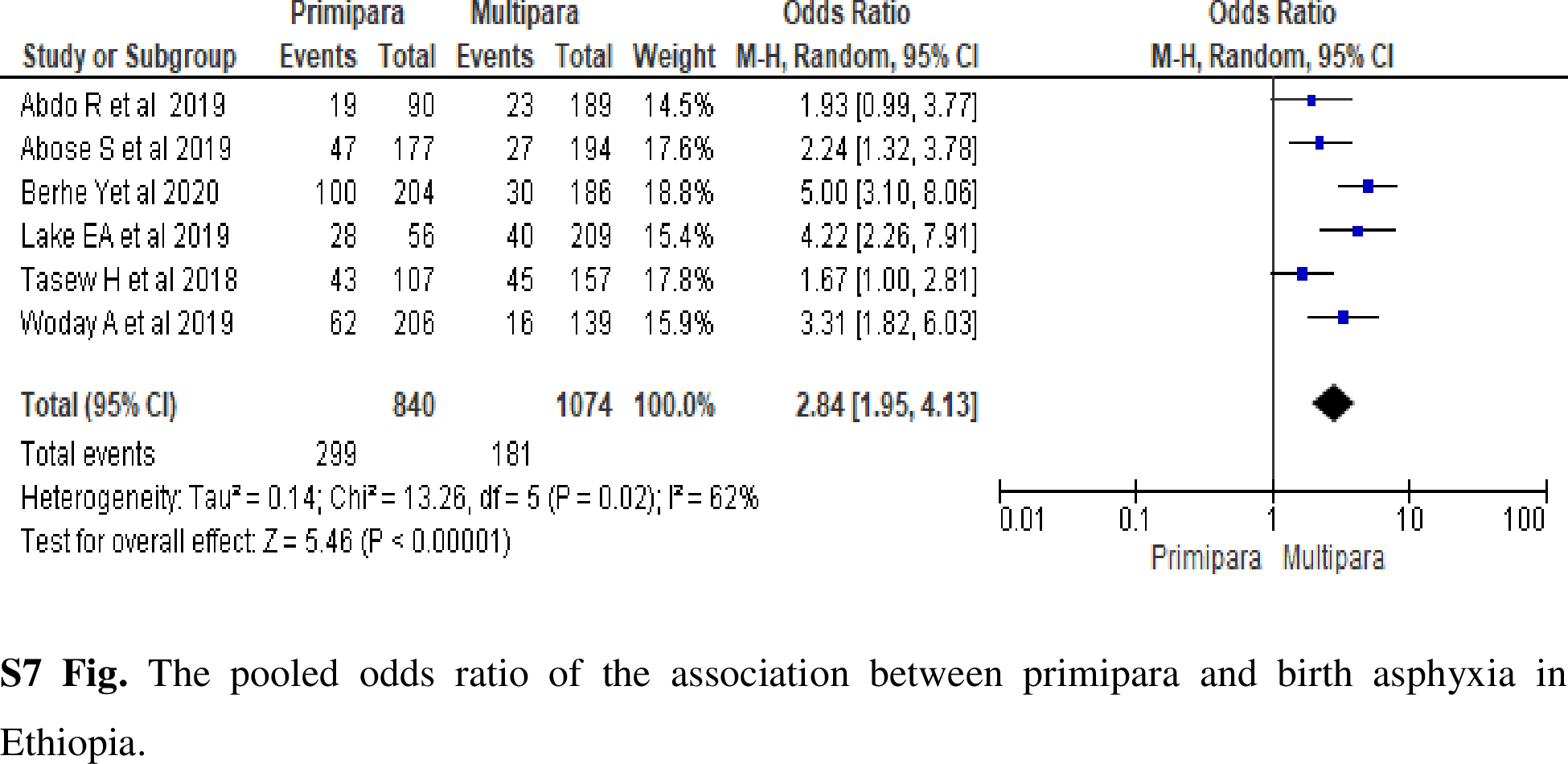

Supplement: S7 Fig — (TIF) [file pone.0255488.s007.tif]

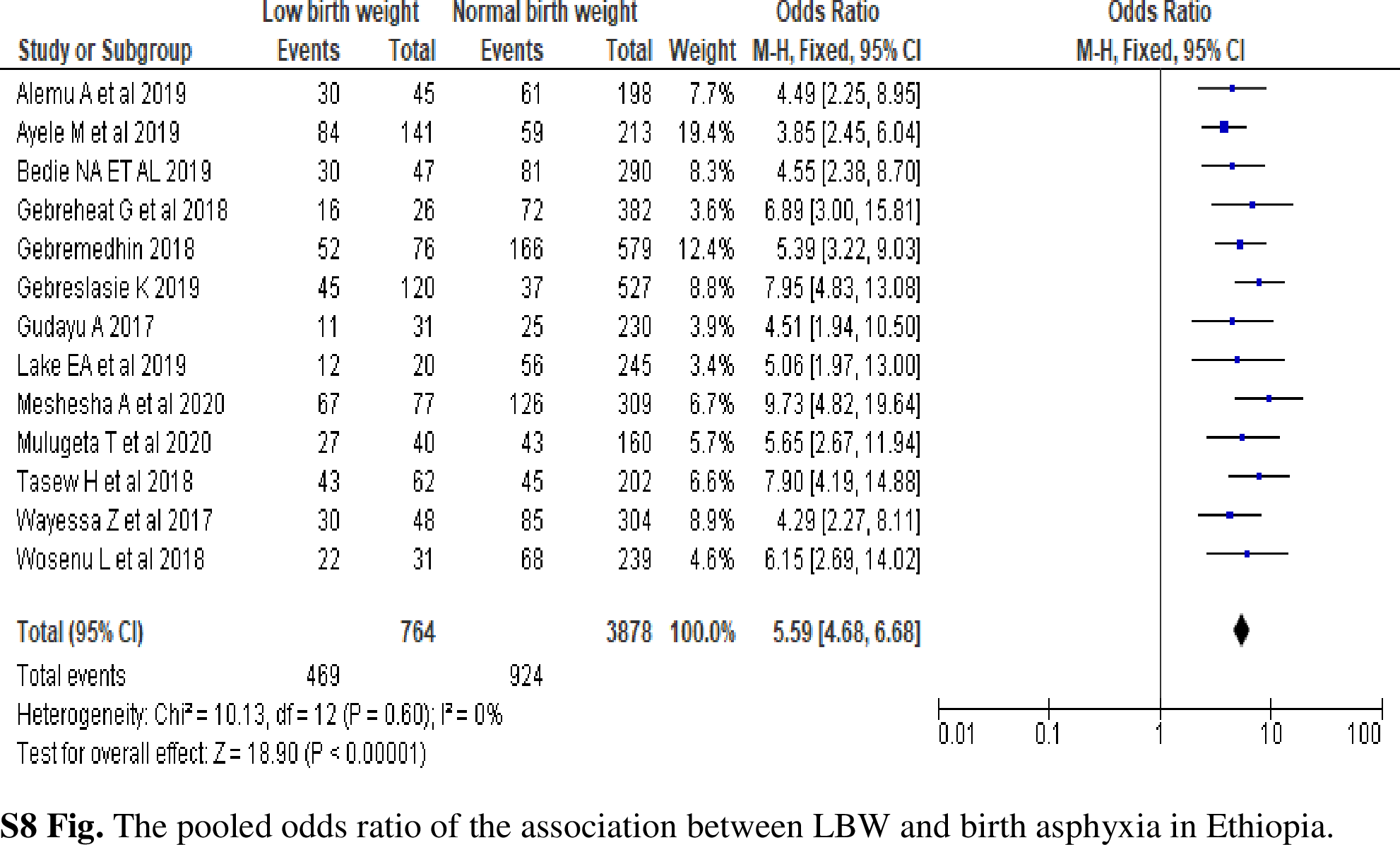

Supplement: S8 Fig — (TIF) [file pone.0255488.s008.tif]

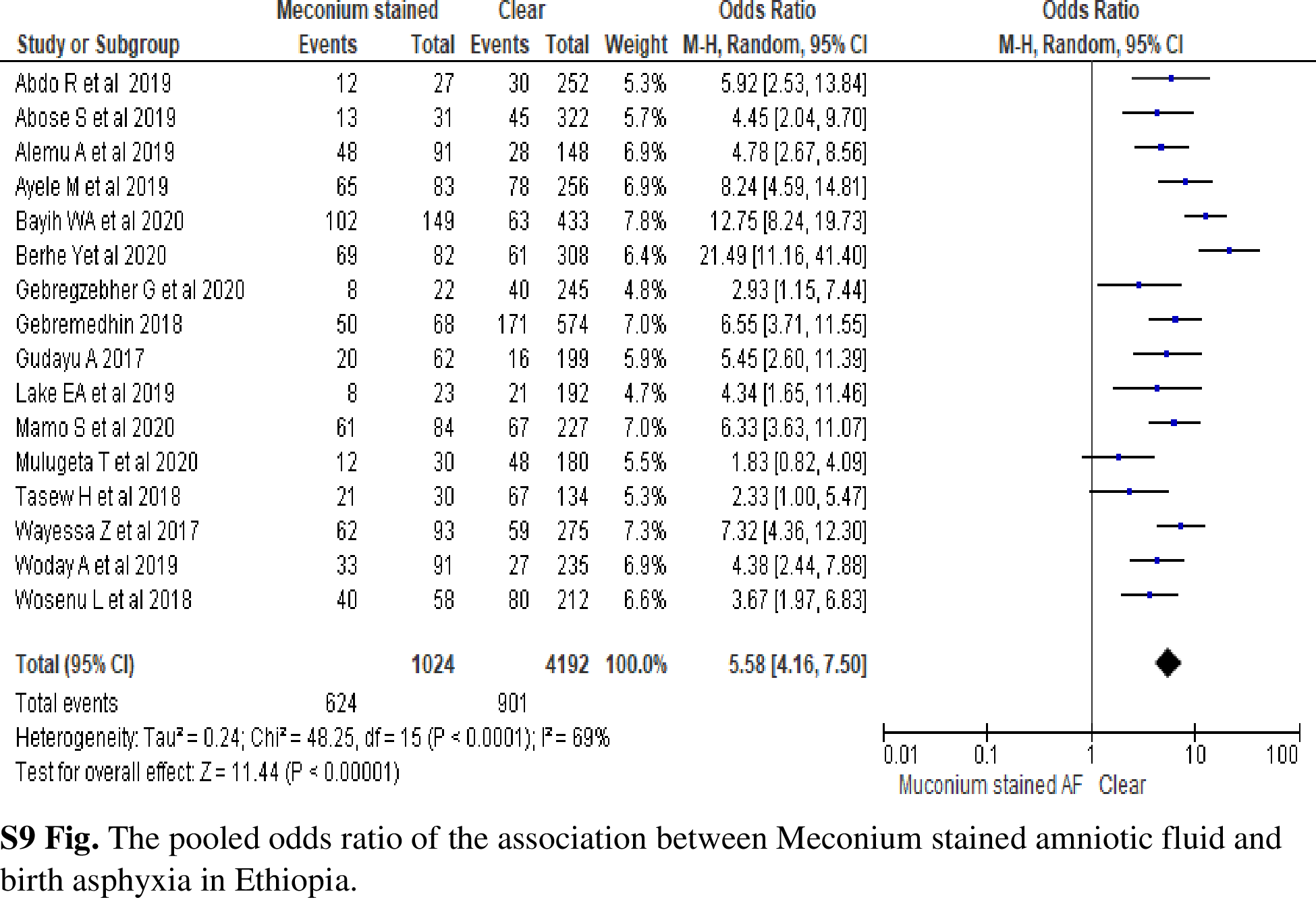

Supplement: S9 Fig — (TIF) [file pone.0255488.s009.tif]

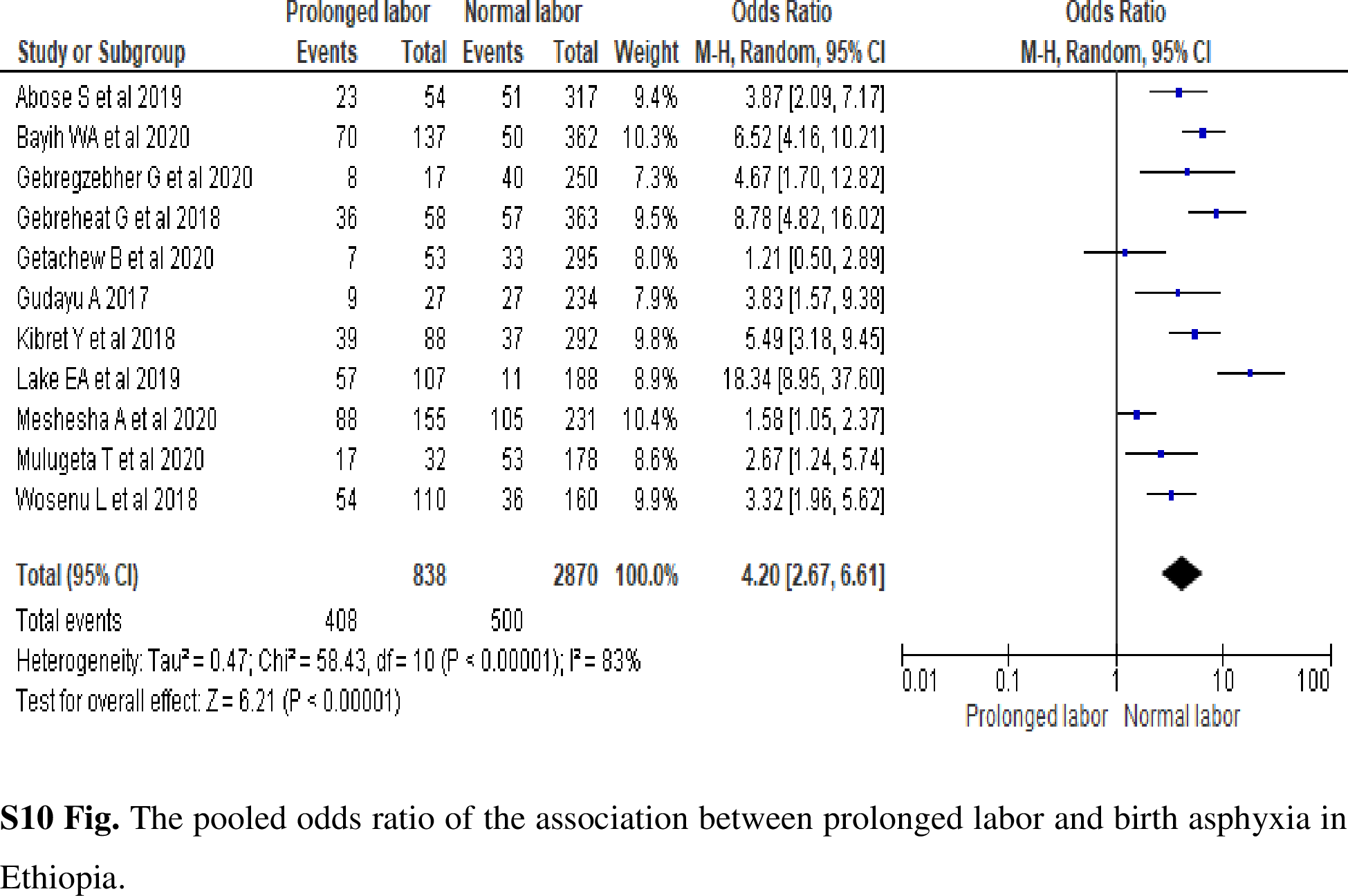

Supplement: S10 Fig — (TIF) [file pone.0255488.s010.tif]

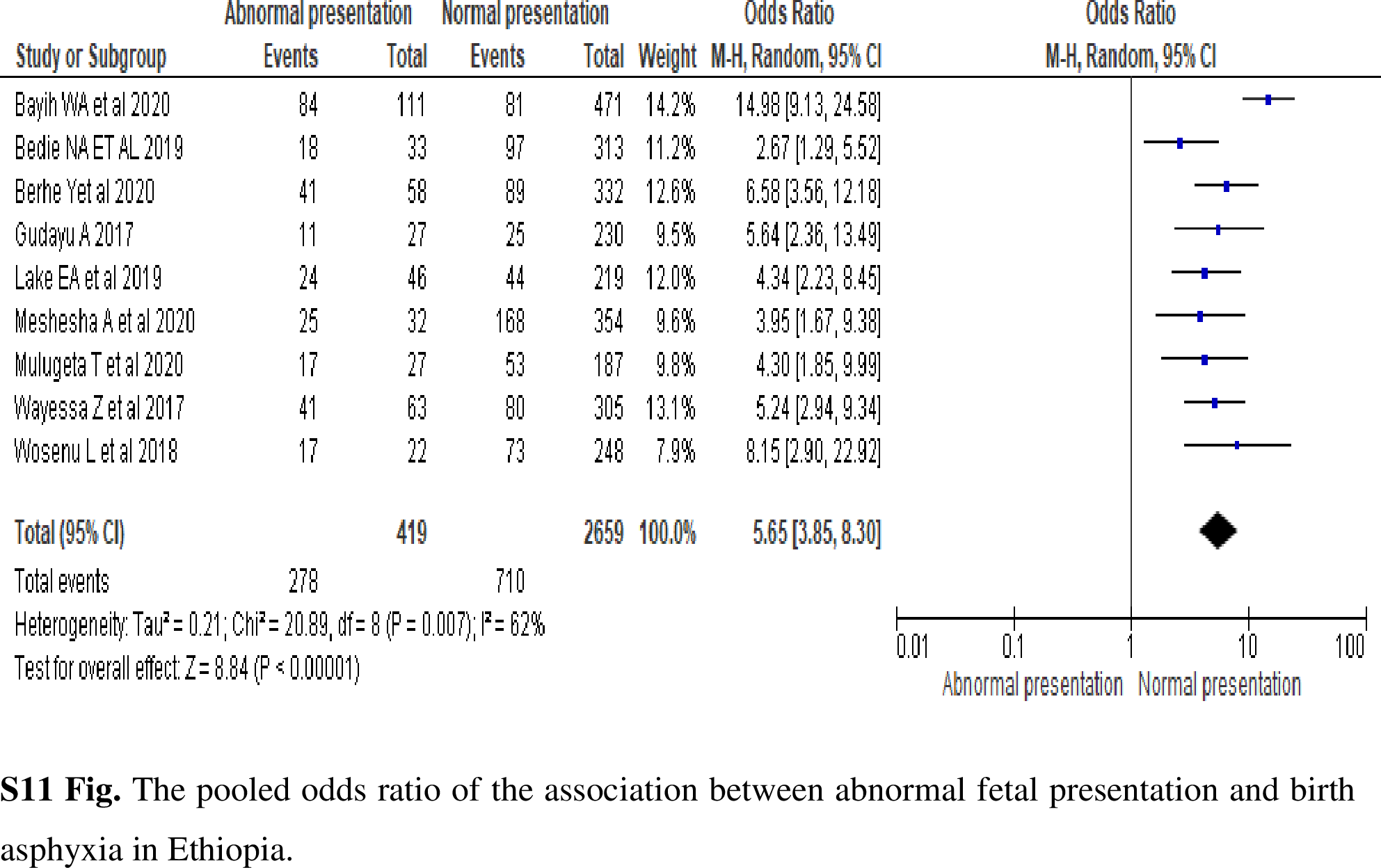

Supplement: S11 Fig — (TIF) [file pone.0255488.s011.tif]

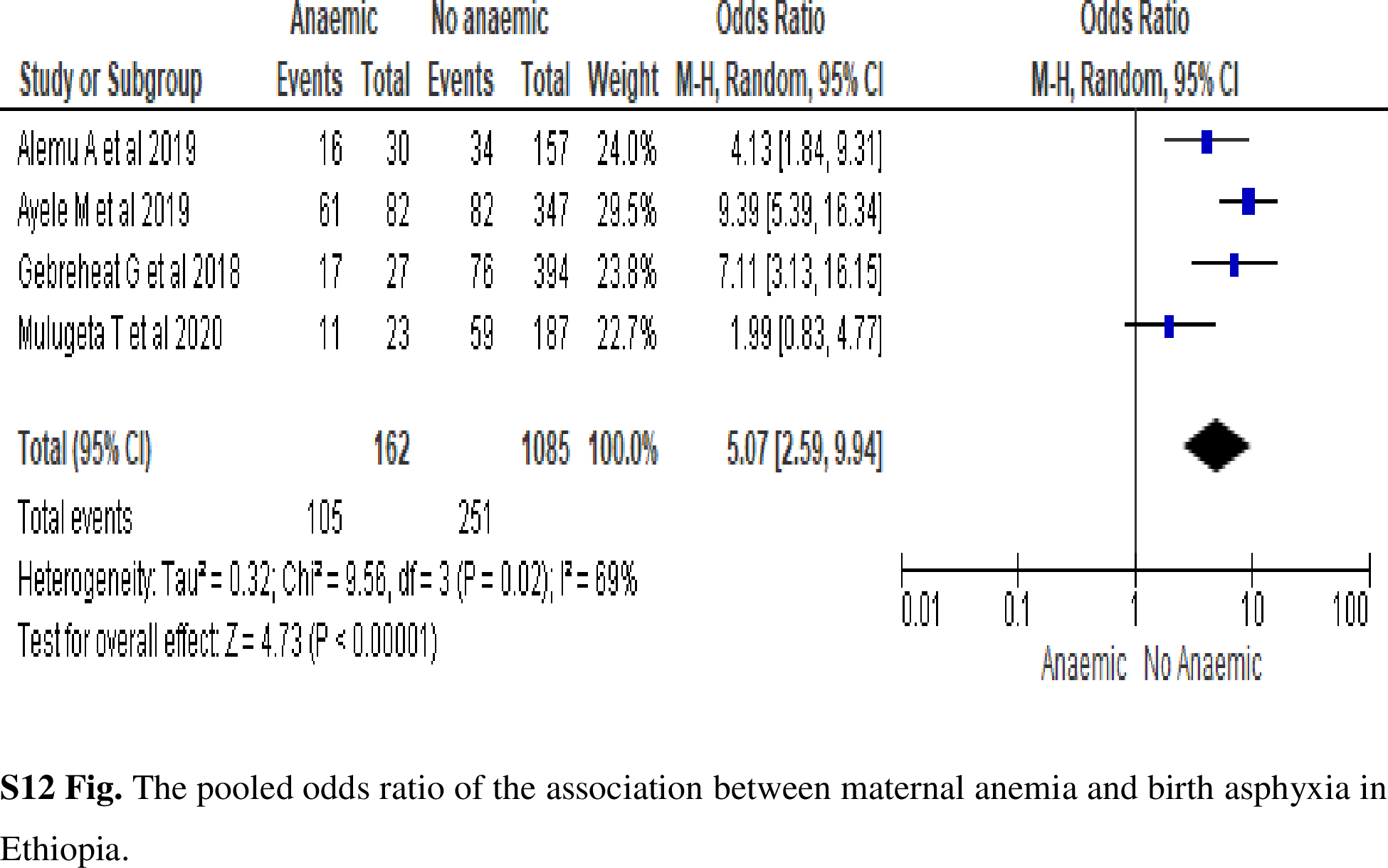

Supplement: S12 Fig — (TIF) [file pone.0255488.s012.tif]

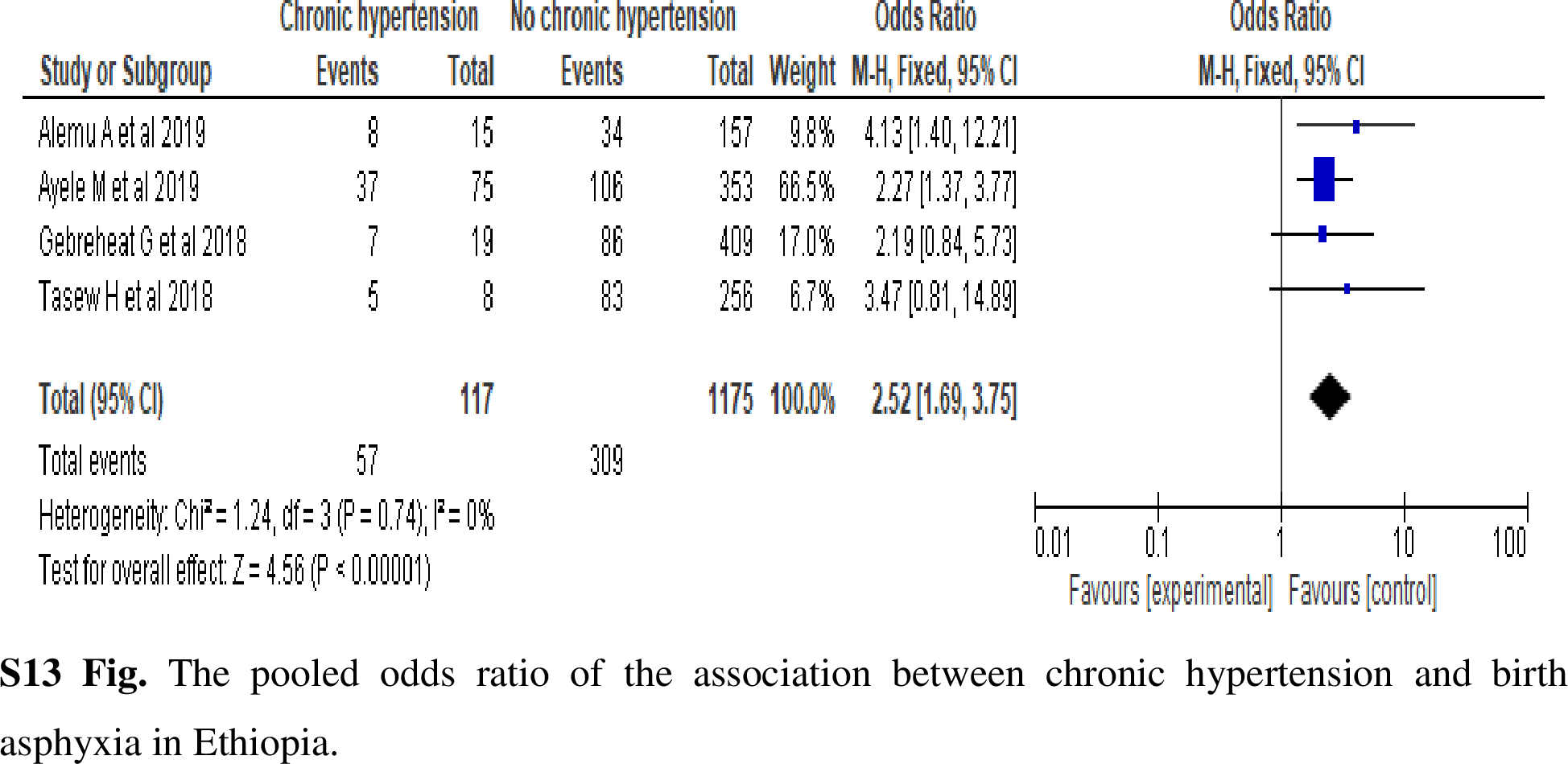

Supplement: S13 Fig — (TIF) [file pone.0255488.s013.tif]

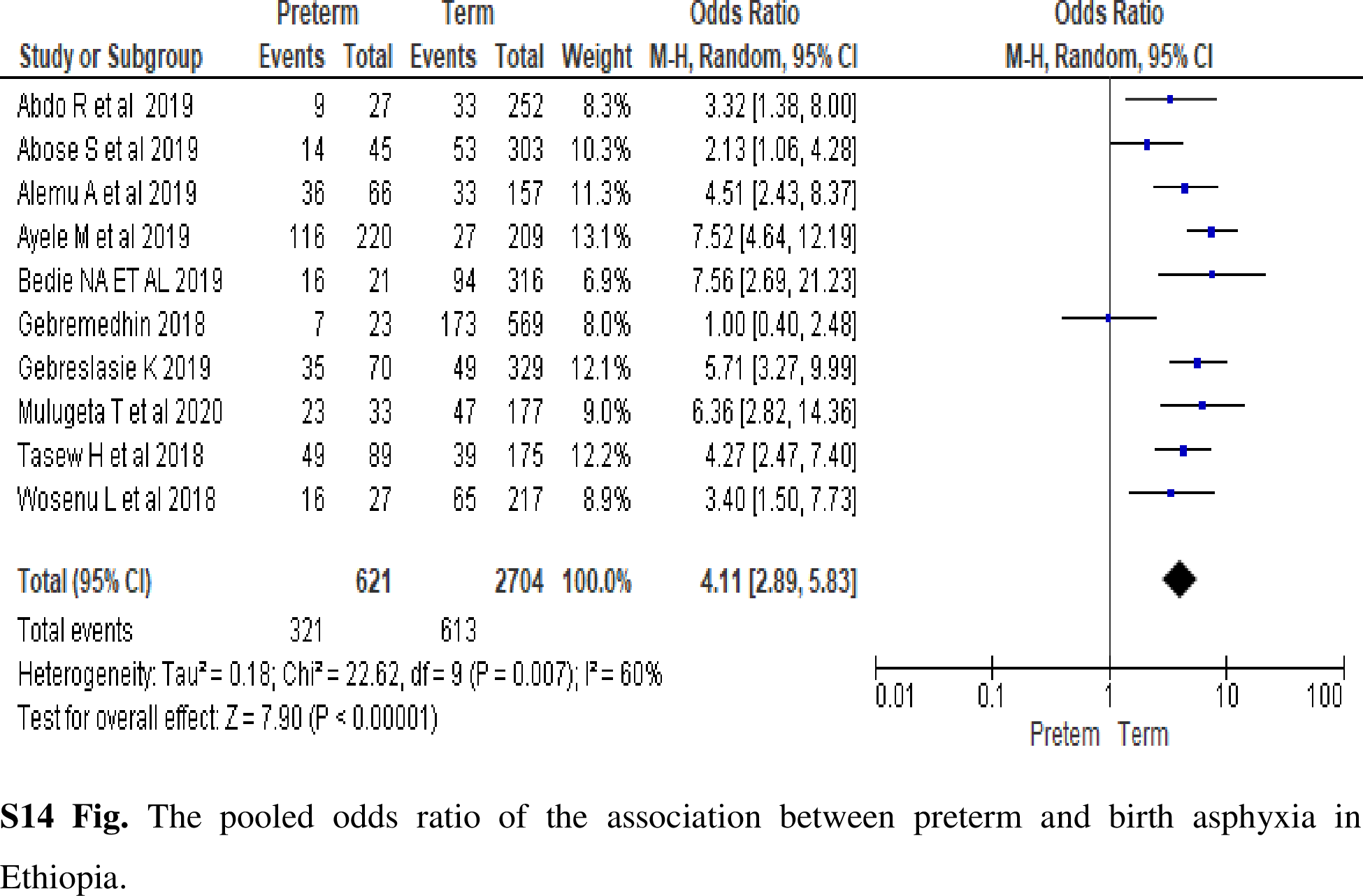

Supplement: S14 Fig — (TIF) [file pone.0255488.s014.tif]

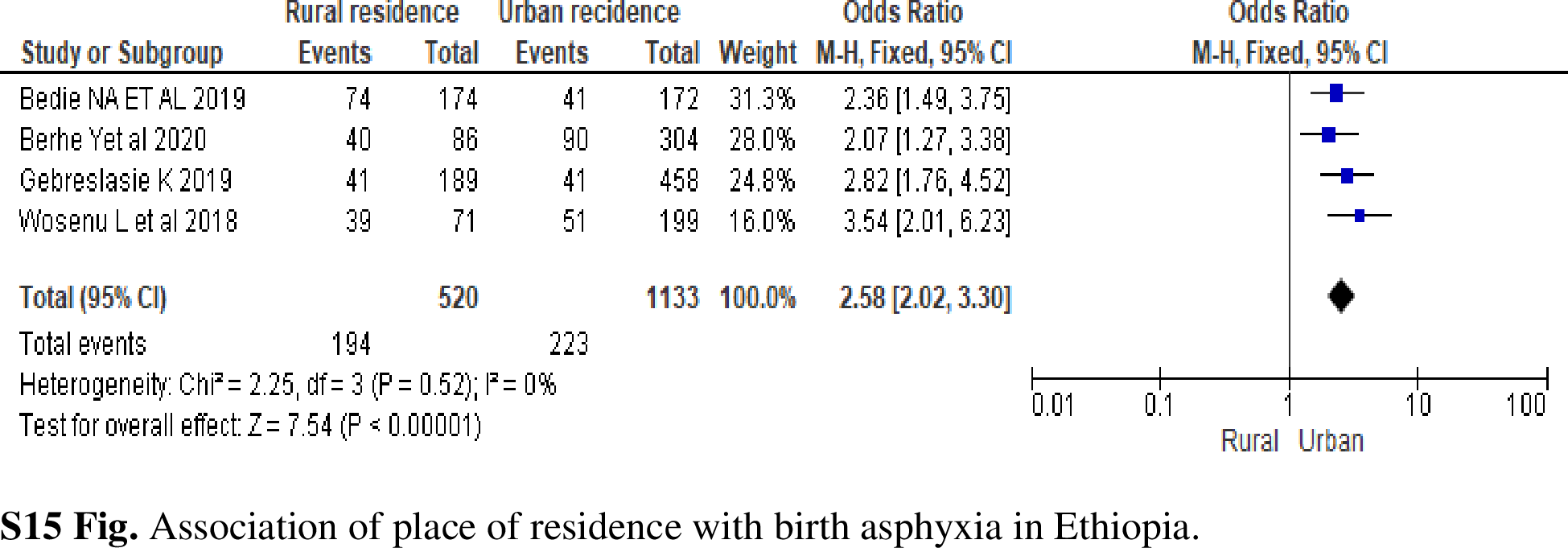

Supplement: S15 Fig — (TIF) [file pone.0255488.s015.tif]

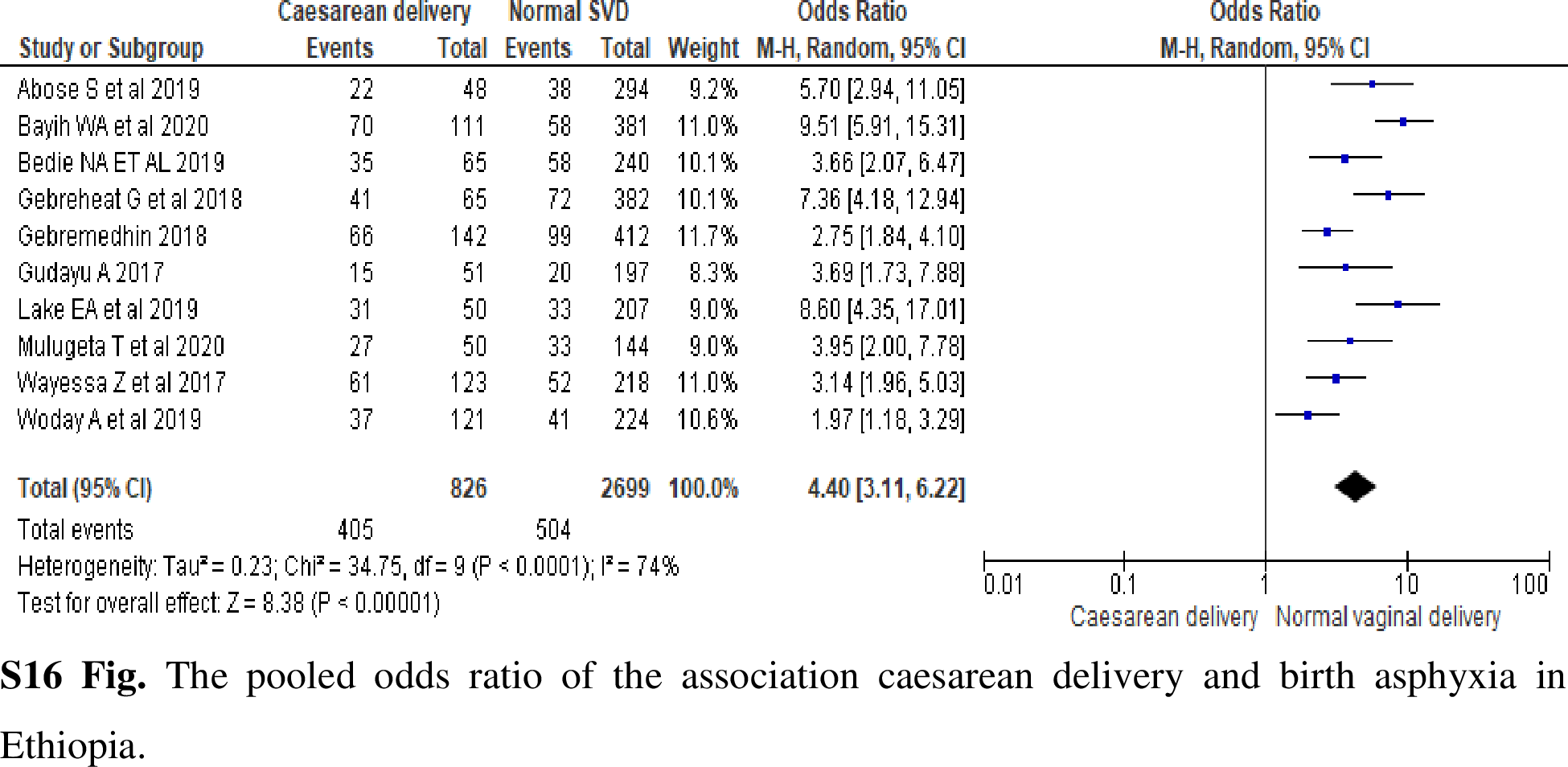

Supplement: S16 Fig — (TIF) [file pone.0255488.s016.tif]

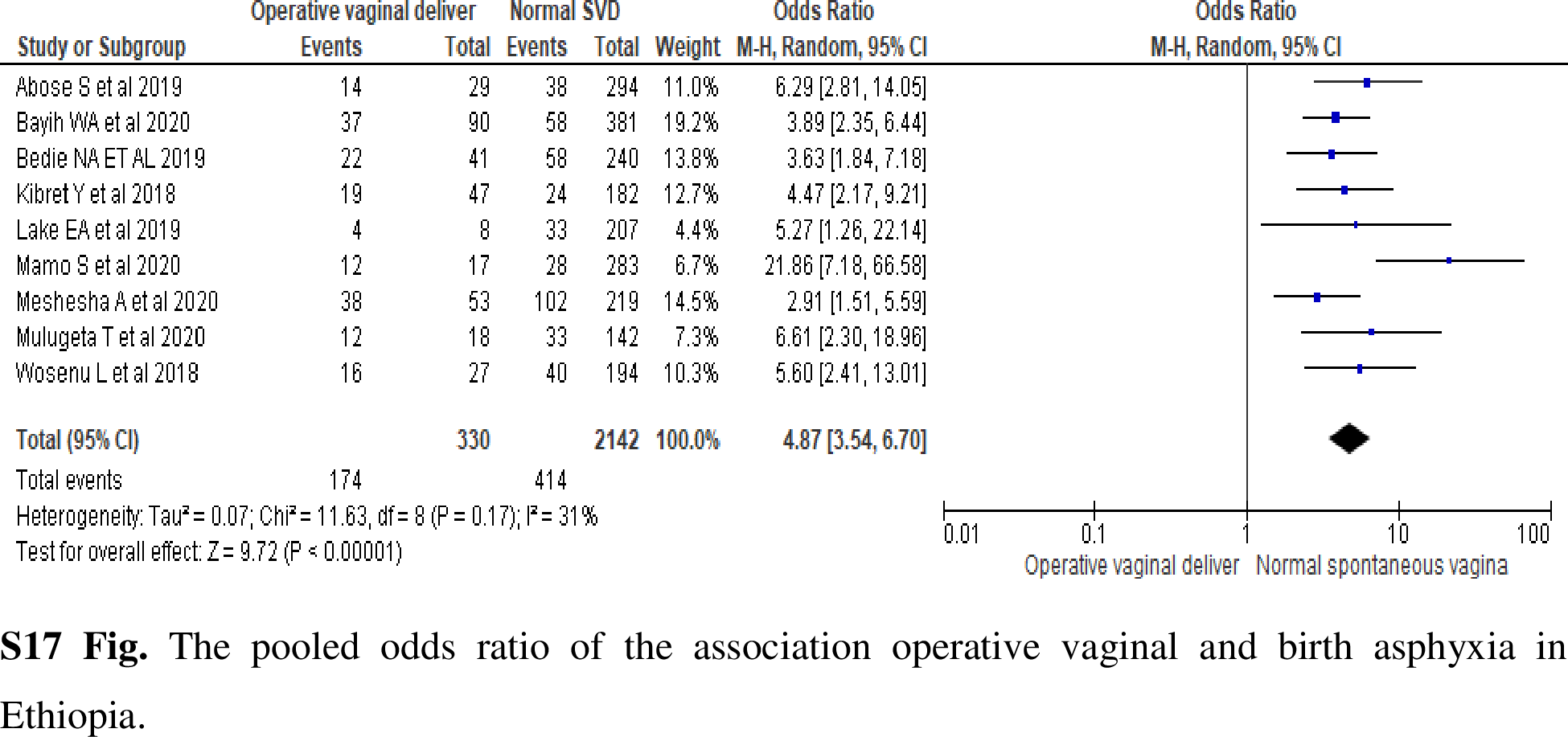

Supplement: S17 Fig — (TIF) [file pone.0255488.s017.tif]

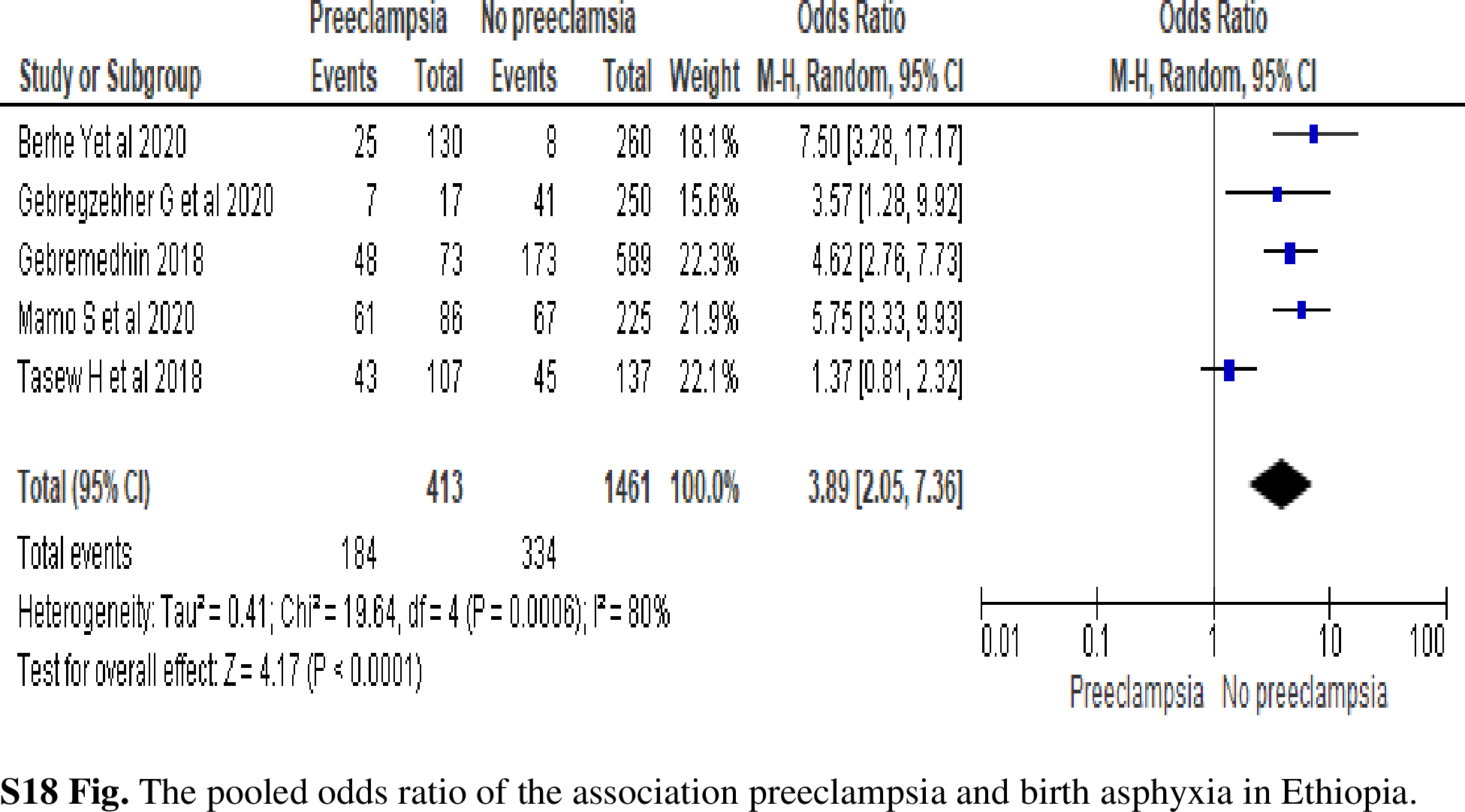

Supplement: S18 Fig — (TIF) [file pone.0255488.s018.tif]

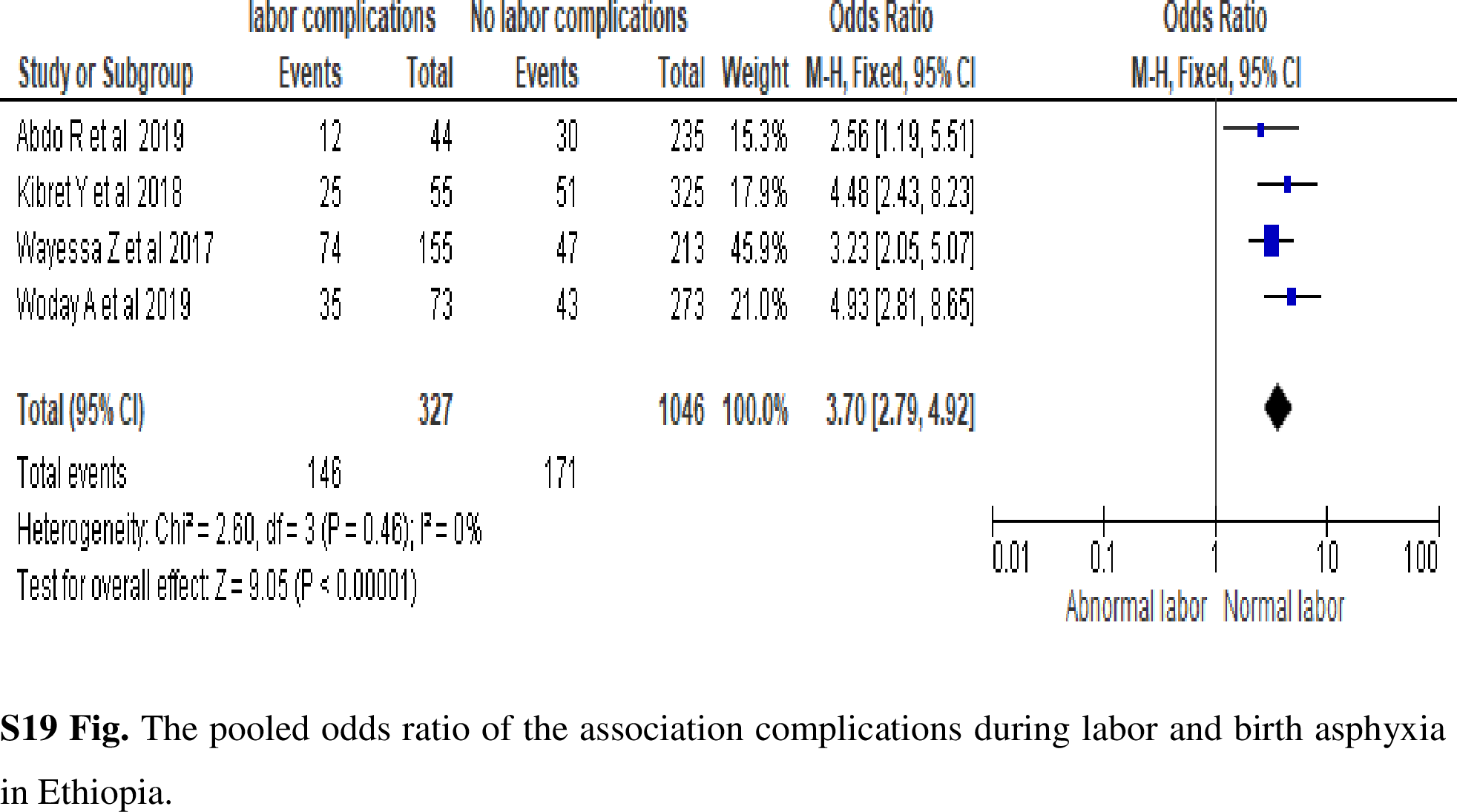

Supplement: S19 Fig — (TIF) [file pone.0255488.s019.tif]

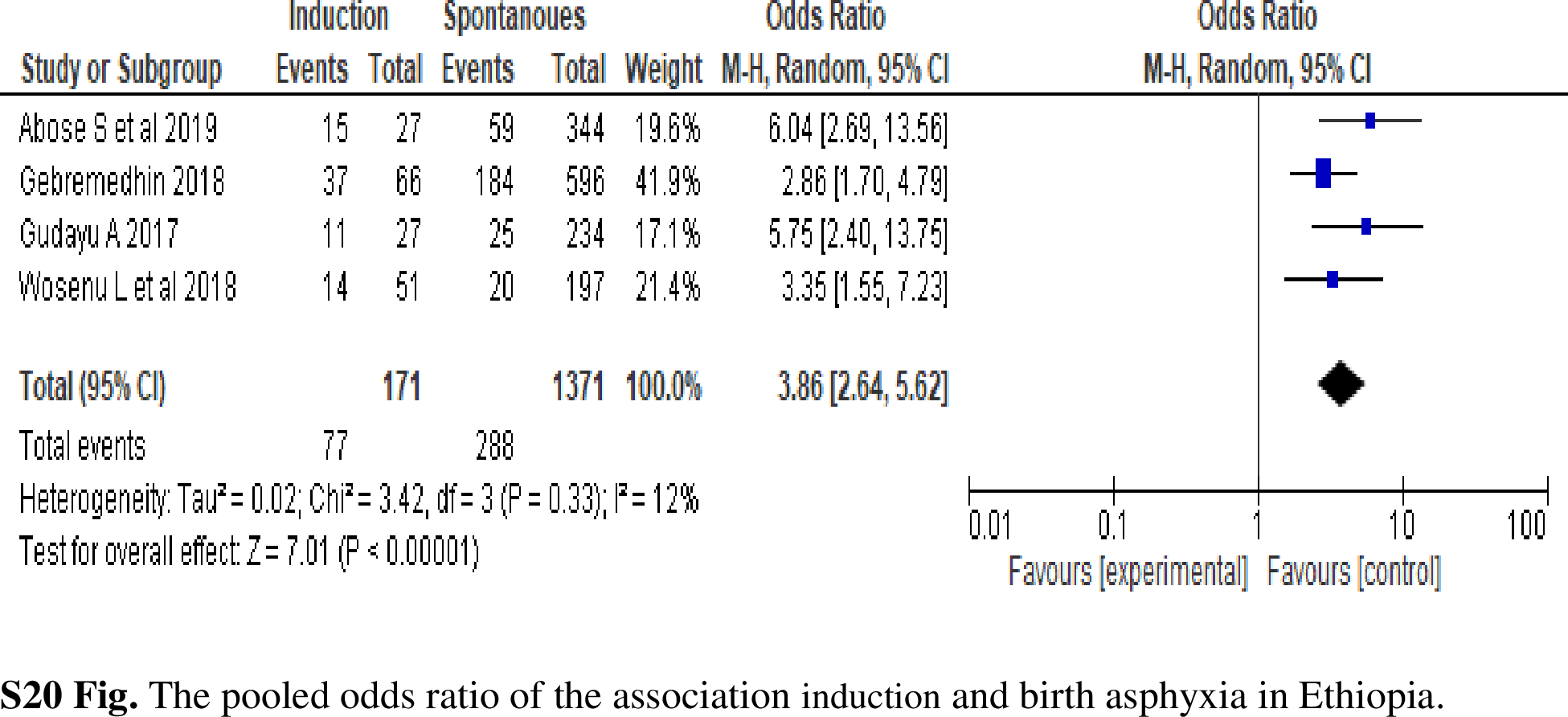

Supplement: S20 Fig — (TIF) [file pone.0255488.s020.tif]

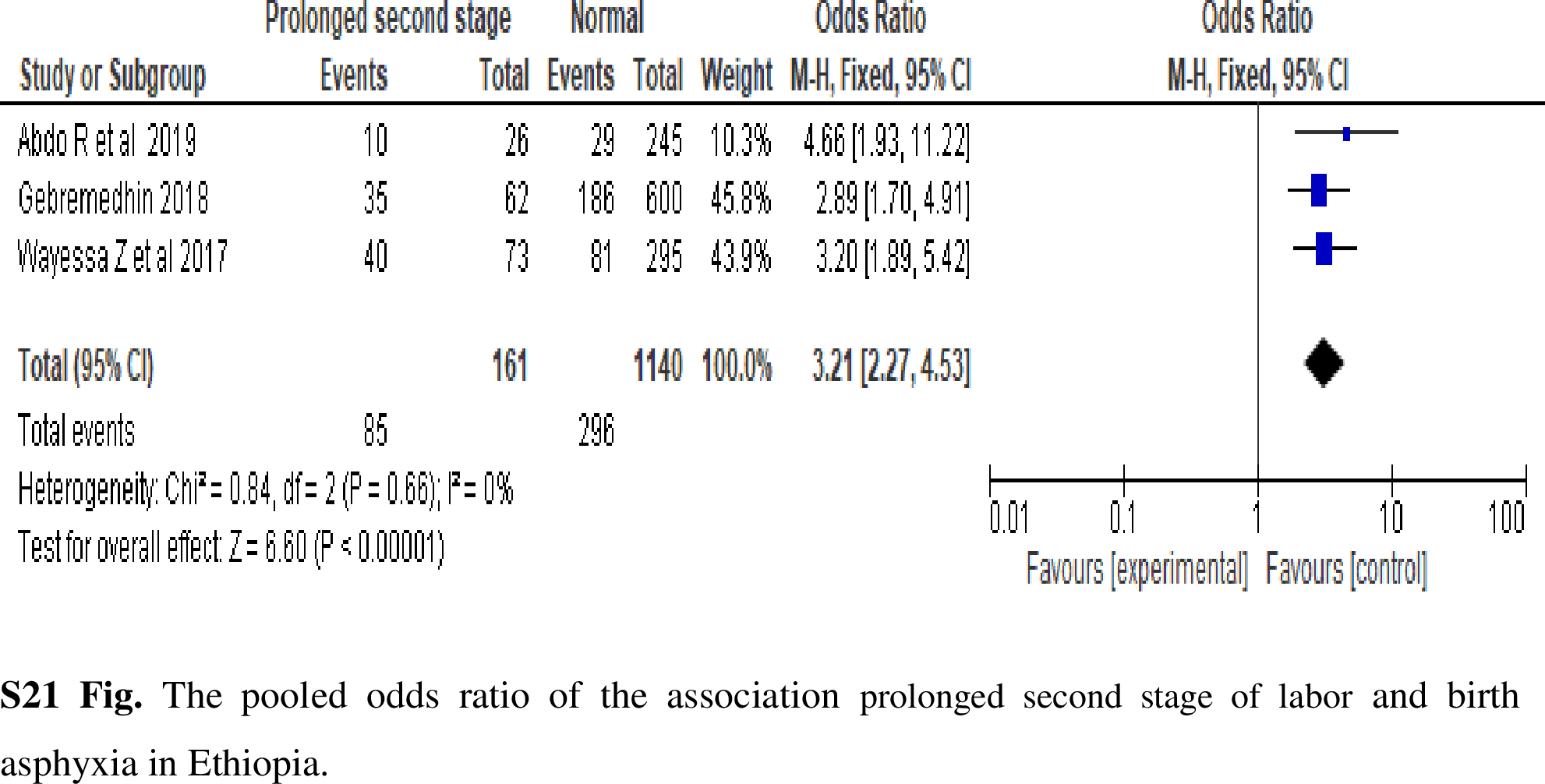

Supplement: S21 Fig — (TIF) [file pone.0255488.s021.tif]

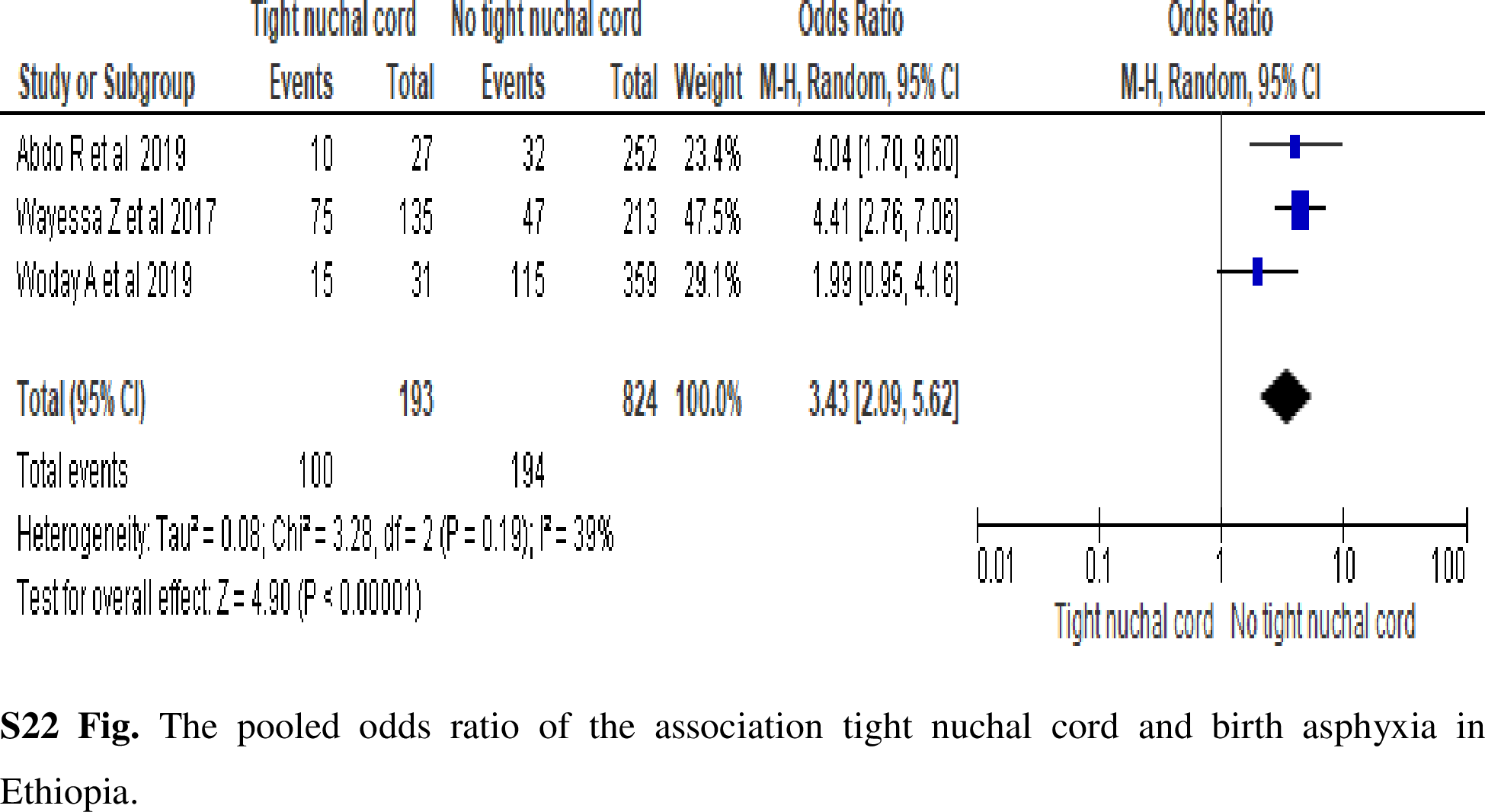

Supplement: S22 Fig — (TIF) [file pone.0255488.s022.tif]

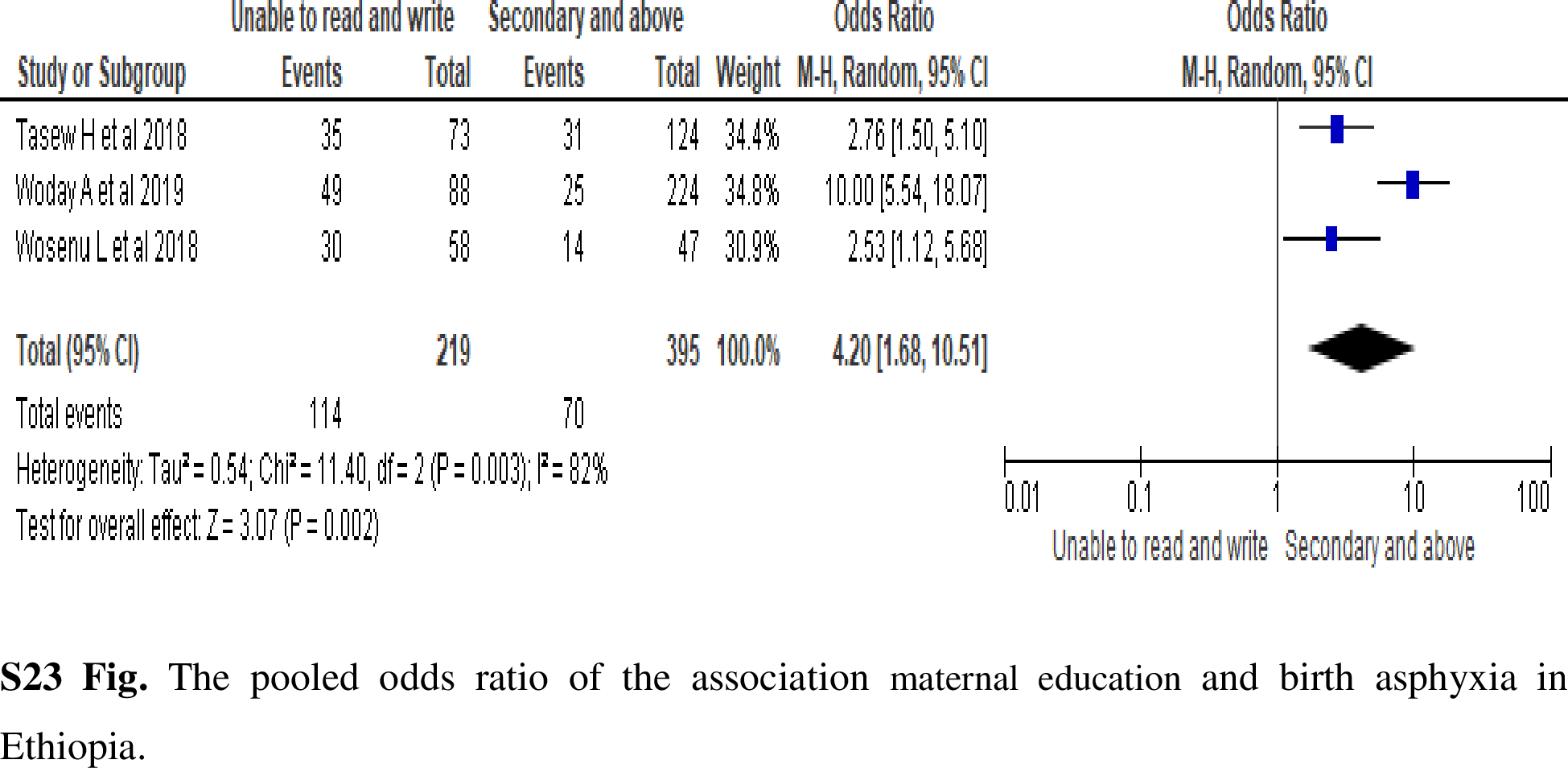

Supplement: S23 Fig — (TIF) [file pone.0255488.s023.tif]

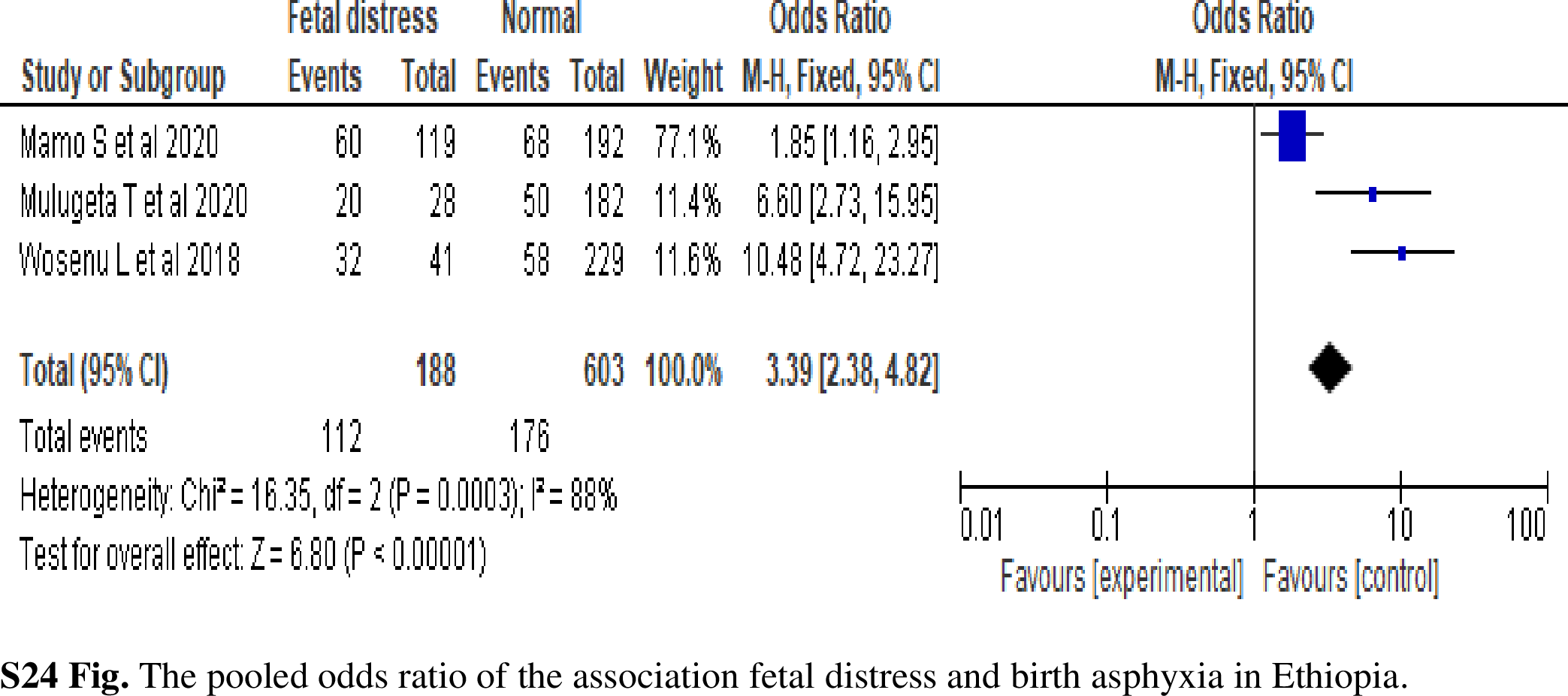

Supplement: S24 Fig — (TIF) [file pone.0255488.s024.tif]

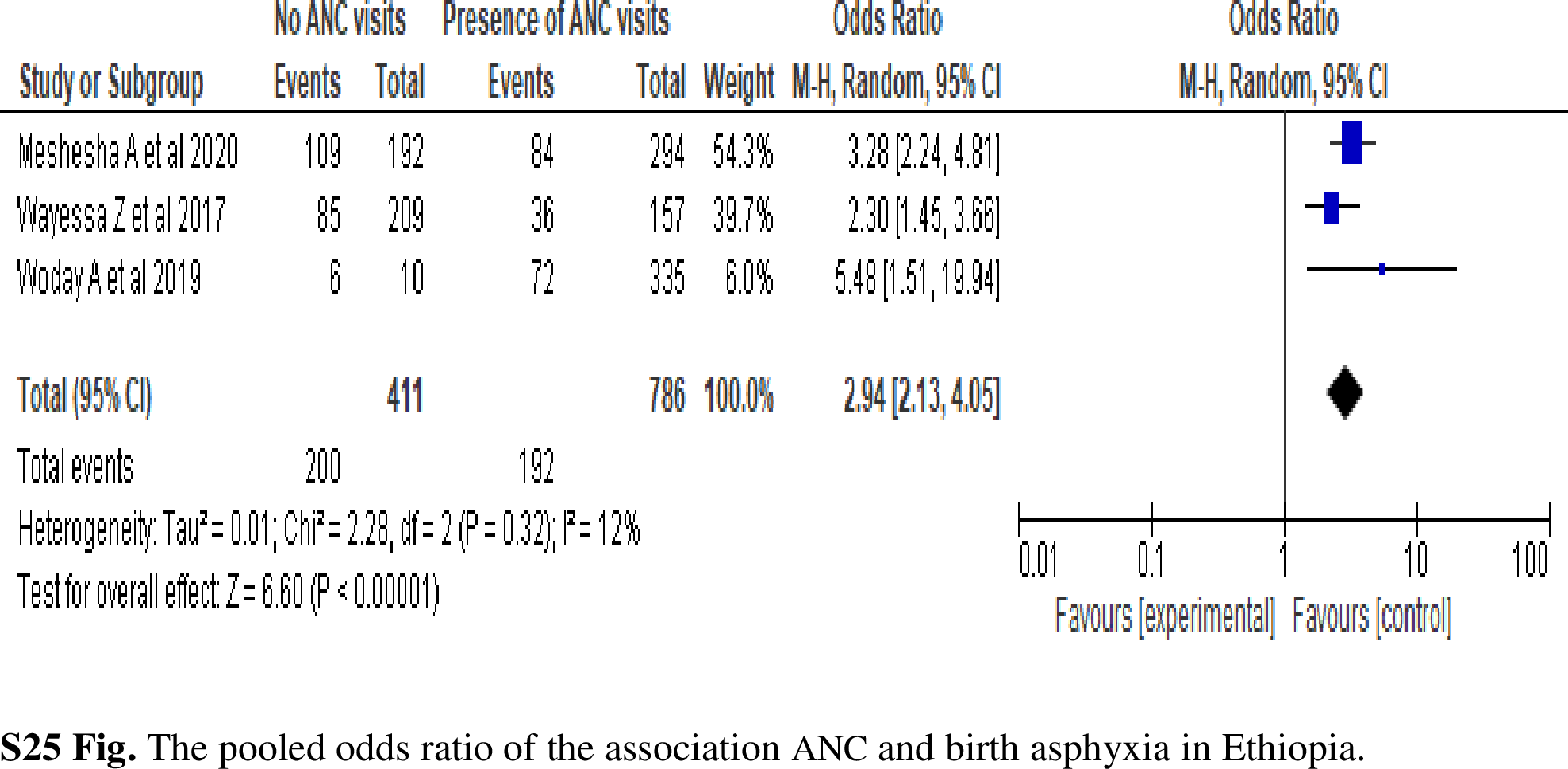

Supplement: S25 Fig — (TIF) [file pone.0255488.s025.tif]

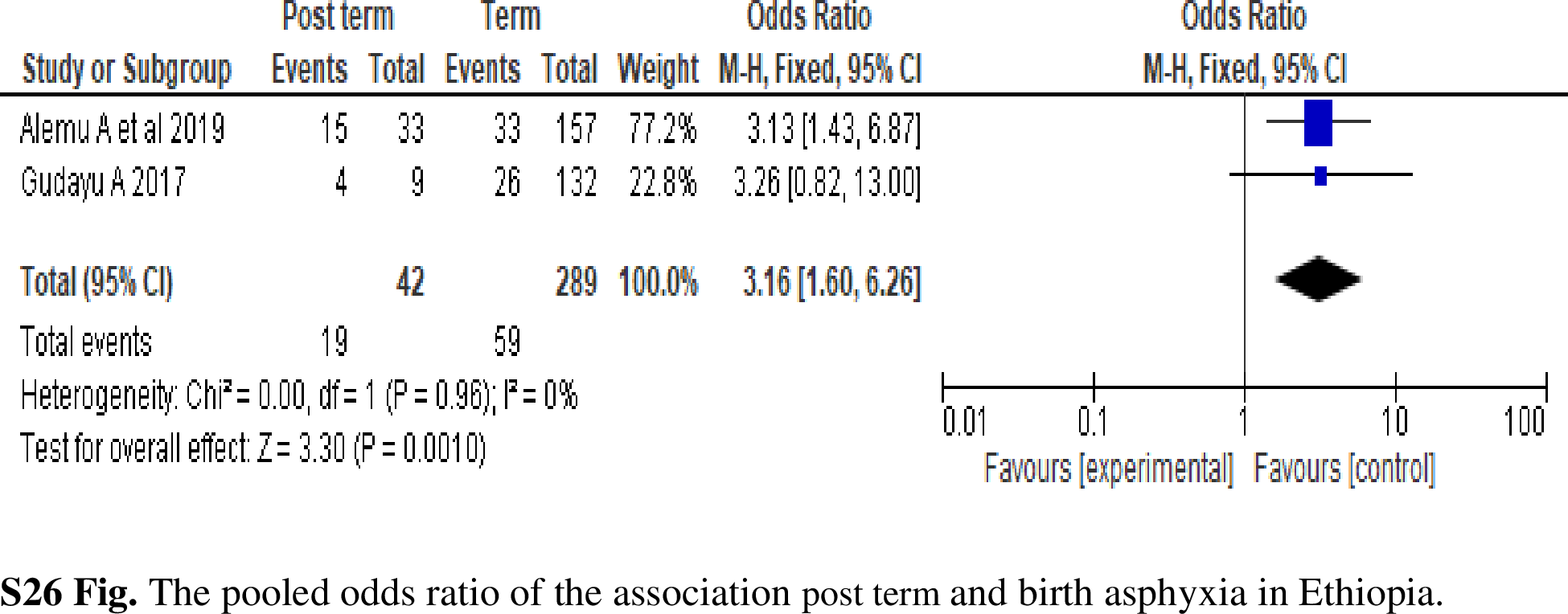

Supplement: S26 Fig — (TIF) [file pone.0255488.s026.tif]
