# Supplementary material for: Prevalence and risk factors associated with birth asphyxia among neonates delivered in Ethiopia: A systematic review and meta-analysis
Source: PLoS One. 2021 Aug 5;16(8):e0255488. doi: 10.1371/journal.pone.0255488 (PMC8341515; doi:10.1371/journal.pone.0255488)
Supplement: S2 File — (DOCX) [file pone.0255488.s028.docx]

**S1 File. Search terms summary.**

| **Database** | **Search terms** | **Results** |
| --- | --- | --- |
| PubMed | (((((((((prevalence[MeSH Terms]) OR prevalence) OR Incidence[MeSH Terms]) OR Incidence) OR Magnitude)) AND ((((((((Risk factors[MeSH Terms]) OR Risk factors) OR Associated) OR Factors) OR Determinants) OR Predictors) OR Correlates) OR Contributing factors)) AND (((((((((((Asphyxia[MeSH Terms]) OR Asphyxia) OR Perinatal asphyxia) OR Apgar score) OR Apgar score[MeSH Terms]) OR hypoxic-ischemic encephalopathy) OR hypoxic-ischemic encephalopathy[MeSH Terms]) OR Birth asphyxia)) OR Asphyxia neonatorum[MeSH Terms]) OR Asphyxia neonatorum)) AND (((((Neonates) OR Infant, Newborn[MeSH Terms]) OR Infant, Newborn)) OR Newborns)) AND ((Ethiopia [MeSH Terms]) OR Ethiopia) | 52 |
| Medline | (((((((((prevalence[MeSH Terms]) OR prevalence) OR Incidence[MeSH Terms]) OR Incidence) OR Magnitude)) AND ((((((((Risk factors[MeSH Terms]) OR Risk factors) OR Associated) OR Factors) OR Determinants) OR Predictors) OR Correlates) OR Contributing factors)) AND (((((((((((Asphyxia[MeSH Terms]) OR Asphyxia) OR Perinatal asphyxia) OR Apgar score) OR Apgar score[MeSH Terms]) OR hypoxic-ischemic encephalopathy) OR hypoxic-ischemic encephalopathy[MeSH Terms]) OR Birth asphyxia)) OR Asphyxia neonatorum[MeSH Terms]) OR Asphyxia neonatorum)) AND (((((Neonates) OR Infant, Newborn[MeSH Terms]) OR Infant, Newborn)) OR Newborns)) AND ((Ethiopia [MeSH Terms]) OR Ethiopia) | 517 |
| Hinari | ((Prevalence) OR (Incidence) OR (Magnitude)) AND ((Associated Factors) OR (Risk factors) OR (Determinants) OR (Predictors)) AND ((Asphyxia) OR (Asphyxia Neonatorum) OR (Birth asphyxia) OR (Perinatal asphyxia) OR (Hypoxic-ischemic encephalopathy) OR (Apgar score)) AND ((Neonates) OR (Newborns) OR (Infant, Newborn)) AND (Delivery) AND (Ethiopia) | 502 |
| Advanced Google Scholar search | Birth asphyxia in Ethiopia 15 | 15 |
|  | Risk factors of birth asphyxia in Ethiopia 3 | 3 |
|  | Perinatal asphyxia in Ethiopia 10 | 10 |
|  | Risk factors of perinatal asphyxia in Ethiopia | 2 |
|  | Determinants of Birth Asphyxia in Ethiopia | 8 |
|  | Determinants of perinatal asphyxia in Ethiopia | 2 |
|  |  |  |
| Others | Grey literature/thesis | 14 |
| Total | | 1,125 |
