# Supplementary material for: Prevalence and risk factors associated with birth asphyxia among neonates delivered in Ethiopia: A systematic review and meta-analysis
Source: PLoS One. 2021 Aug 5;16(8):e0255488. doi: 10.1371/journal.pone.0255488 (PMC8341515; doi:10.1371/journal.pone.0255488)
Supplement: S3 File — (DOCX) [file pone.0255488.s029.docx]

**S2 file. Quality appraisal of included study**

| Included studies | **Eight JBI Critical Appraisal Checklist for Cross Sectional Studies(Yes,No,Unclear)**  **If Yes(>=50%).....low risk(two raters).....>1=Yes 0=No/Unclear** | | | | | | | | | | | | | | | | |
| --- | --- | --- | --- | --- | --- | --- | --- | --- | --- | --- | --- | --- | --- | --- | --- | --- | --- |
|  | Q1 | | Q2 | | Q3 | | Q4 | | Q5 | | Q6 | | Q7 | | Q8 | | Over all appraisal |
|  | R1 | R2 | R1 | R2 | R1 | R2 | R1 | R2 | R1 | R2 | R1 | R2 | R1 | R2 | R1 | R2 |  |
| 1. Gebreheat G et all | Y | Y | Y | Y | Y | Y | Y | Y | Y | N | Y | Y | Y | Y | Y | U | 7 |
| 1. Gudayu TW. | Y | Y | Y | Y | Y | Y | Y | N | N | N | N | N | Y | Y | Y | Y | 5.5 |
| 1. Woday A. et al | Y | Y | Y | Y | Y | Y | Y | Y | Y | Y | Y | Y | Y | Y | Y | Y | 8 |
| 1. G/ziabher GT. et al | Y | Y | Y | Y | Y | Y | Y | Y | Y | U | Y | Y | Y | Y | Y | Y | 7.5 |
| 1. Abebe A. et al | Y | Y | U | U | Y | Y | Y | Y | Y | Y | Y | Y | Y | Y | Y | Y | 7 |
| 1. Abdo RA et al. | Y | N | U | N | Y | Y | Y | Y | Y | Y | Y | Y | Y | Y | Y | Y | 6.5 |
| 1. Salamu A et al | N | N | N | N | Y | Y | Y | Y | Y | U | Y | Y | Y | Y | Y | Y | 5.5 |
| 1. Gebreslasie K et al | N | Y | N | Y | Y | Y | Y | Y | Y | U | Y | Y | Y | Y | Y | Y | 6.5 |
| 1. Ibrahim NA et al | Y | Y | Y | Y | Y | Y | Y | Y | N | U | N | U | Y | Y | Y | Y | 6 |
| 1. Getachew B et al | Y | Y | Y | Y | Y | Y | Y | Y | Y | N | Y | U | Y | Y | Y | Y | 7 |
| 1. Wayessa ZJ et al | Y | Y | Y | Y | Y | Y | Y | Y | Y | Y | Y | Y | Y | Y | Y | U | 7.5 |
| 1. Jamie AH | Y | Y | N | U | Y | Y | Y | Y | Y | Y | Y | Y | Y | Y | Y | U | 6.5 |
| 1. Demisse AG | Y | Y | U | U | Y | Y | Y | N | N | Y | Y | Y | Y | Y | Y | Y | 6 |
| 1. Mamo SA et al | N | U | N | U | Y | Y | Y | Y | Y | Y | Y | Y | Y | Y | Y | U | 5.5 |
| 1. Asfere NW 2018 | Y | Y | Y | Y | Y | U | Y | Y | N | N | Y | Y | N | N | N | N | 4.5 |
| 1. Bayih WA et al 2020 | Y | Y | U | U | Y | Y | Y | Y | N | N | Y | Y | Y | Y | Y | Y | 6 |
| 1. Lake EA et al 2019 | N | N | U | U | Y | Y | Y | Y | N | N | Y | Y | Y | Y | N | N | 4 |

| Included studies | Ten JBI Critical Appraisal Checklist for Case control Studies(Yes, No, Unclear)  If Yes(>=50%).....low risk(two raters).....>1=Yes 0=No/Unclear | | | | | | | | | | | | | | | | | | | | |
| --- | --- | --- | --- | --- | --- | --- | --- | --- | --- | --- | --- | --- | --- | --- | --- | --- | --- | --- | --- | --- | --- |
|  | Q1 | | Q2 | | Q3 | | Q4 | | Q5 | | Q6 | | Q7 | | Q8 | | Q9 | | Q10 | | Over all |
|  | 1 | R2 | R1 | R2 | R1 | R2 | R1 | R2 | R1 | R2 | R1 | R2 | R1 | R2 | R1 | R2 | R1 | R2 | R1 | R2 |  |
| 1. Ayele MW et al | Y | Y | Y | N | Y | Y | Y | Y | Y | Y | N | N | N | N | N | Y | Y | Y | Y | Y | 7 |
| 1. Wosenu L et al | Y | N | Y | Y | Y | Y | Y | Y | Y | Y | Y | Y | Y | U | Y | U | Y | Y | Y | Y | 8.5 |
| 1. Meshesha AD et al | Y | Y | Y | Y | Y | Y | Y | Y | Y | Y | N | Y | N | Y | Y | Y | Y | Y | Y | Y | 9 |
| 1. Kibret Y et al | Y | Y | Y | N | Y | Y | N | Y | Y | Y | N | U | N | U | Y | U | Y | Y | Y | Y | 6.5 |
| 1. Berhe YZ | Y | Y | Y | Y | Y | U | Y | Y | Y | Y | N | Y | N | Y | Y | Y | Y | Y | Y | Y | 8.5 |
| 1. Tasew H et al. | Y | Y | Y | N | Y | Y | Y | Y | Y | Y | N | U | N | U | Y | Y | Y | U | Y | Y | 7 |
| 1. Mulugeta T | Y | Y | Y | Y | Y | Y | N | Y | Y | Y | N | U | N | U | Y | Y | Y | Y | Y | Y | 7.5 |
| 1. Gebremedhin MM et al 2018 | Y | Y | Y | Y | Y | Y | Y | Y | Y | Y | Y | Y | Y | Y | Y | Y | Y | Y | Y | Y | 10 |
| 1. Bedie NA et al 2019 | Y | Y | N | N | Y | Y | N | N | N | N | N | N | N | Y | Y | Y | Y | Y | Y | Y | 5 |
